# Joint impact of stress hyperglycaemic ratio and glycaemic variability in patients with ischaemic stroke and machine learning for mortality prediction

**DOI:** 10.1186/s12883-025-04510-z

**Published:** 2025-11-25

**Authors:** Linting Gu, Sheng Chen, Zhenkun Yang, Yang Liu, Ziyi Zhong, Yang Chen

**Affiliations:** 1 Department of Neurology, Shanghai Public Health Clinical Center, Fudan University, Shanghai, People’s Republic of China; 2 Department of Nephrology, China-Japan Friendship Hospital, Beijing, People’s Republic of China; 3 China-Japan Friendship Hospital (institute of Clinical Medical Sciences), Chinese academy of Medical Sciences & Peking union Medical College, Beijing, 100029 China; 4 Liverpool Centre for Cardiovascular Science, University of Liverpool, Liverpool John Moores University and Liverpool Heart and Chest Hospital, William Henry Duncan Building, 6 West Derby Street, Liverpool, L7 8TX UK; 5 Department of Cardiovascular Medicine, the Second Affiliated Hospital, Jiangxi Medical College, Nanchang University, Nanchang, Jiangxi People’s Republic of China; 6 Department of Musculoskeletal Ageing and Science, Institute of Life Course and Medical Sciences, University of Liverpool, Liverpool, UK; 7 Department of Cardiovascular and Metabolic Medicine, Institute of Life Course and Medical Sciences, University of Liverpool, Liverpool, UK

**Keywords:** Ischaemic stroke, Stress hyperglycemia ratio, Glycaemic variability, Intensive care unit, Mortality, Machine learning

## Abstract

**Background:**

The global burden of ischaemic stroke (IS) is high, which is potentially relevant to stress hyperglycemia ratio (SHR) and glycaemic variability (GV). This study aims to evaluate the combined effect of the SHR and GV with predict short-term, medium-term and long-term mortality outcomes in the intensive care unit (ICU).

**Methods:**

This retrospective study utilised data from the MIMIC-IV database, including adult ICU patients diagnosed with ischaemic stroke. SHR and GV were calculated and categorised into tertiles, with combined effects grouped into four categories. Study outcomes included 30-day, 90-day, and 360-day mortality outcomes. Kaplan-Meier curves, restricted cubic splines and Cox proportional hazards models were used to assess the SHR and GV with mortality outcomes. Then, we further assessed the associations with subgroup analyses by diabetes, age, sex, and body mass index. Predictive performance was evaluated using receiver operating characteristic curves and area under the curve (AUC) comparisons.

**Results:**

In 749 patients with IS (age 72.9 [61.1–83.0] years; 47.3% male), and 30-day, 90-day, and 360-day ICU mortality rates of 23.2%, 29.6%, and 35.4%, respectively. Patients with both high SHR and high GV (G4 vs. G1) had the highest mortality risk in the overall population, with HRs of 2.43 (95% CI: 1.42–4.14) for 30-day mortality, 2.18 (95% CI: 1.36–3.06) for 90-day mortality, and 1.77 (95% CI: 1.14–2.74) for 360-day mortality. The combined effect of SHR and GV demonstrated superior predictive performance (AUC: 0.643 for 30-day, 0.652 for 90-day, and 0.640 for 360-day mortality) compared to SHR or GV alone. These findings highlight the prognostic utility of combining SHR and GV for mortality prediction in critically ill patients with IS.

**Conclusion:**

The combination of SHR and GV is promising to facilitate early identifying IS critically ill patients at high-risk of mortality.

**Supplementary Information:**

The online version contains supplementary material available at 10.1186/s12883-025-04510-z.

## Introduction

Ischaemic stroke (IS) is a condition where there is a blockage in the blood circulation pathway, typically caused by a clot, restricting the flow of blood to a specific region of the brain. Despite ongoing efforts in disease prevention and treatment, IS remains one of the major causes of mortality and morbidity around the world [1]. Latest Global Burden of Disease Study suggests about 7.63 (6.57–8.96) million IS cases globally in 2019, causing 11.6% of total deaths and 5.7% of total disability-adjusted life-years [2]. Recent study suggested that the 30-day mortality for patients with IS in the intensive care unit (ICU) was over 30% [3]. Therefore, it is imperative to find potentially strong predictors to guide mortality risk stratification for IS in the ICU.

Currently high fasting plasma glucose is one of the top five risk factors for stroke [2]. The rapidly increasing prevalence of glycemic abnormalities and diabetes exacerbates poor cerebrovascular outcomes in IS patients [4]. Elevated blood glucose levels, known as hyperglycemia, are frequently observed in critically ill patients, with about 50% of ICU admissions experiencing this condition within the first 48 h [5]. Stress-induced hyperglycemia induces insulin resistance, resulting in increased blood sugar levels.

Stress hyperglycemia ratio (SHR), a measure introduced by the Roberts group, is widely acknowledged for its role in identifying stress hyperglycemia [6]. SHR is regarded as a more predictive prognostic marker in critically ill patients. Several recent studies have consistently shown that SHR correlates with unfavorable prognosis in patients with IS, including heightened probabilities of functional disabilities, stroke reappearance, and mortality [7–9]. Nevertheless, the SHR calculation solely depends on initial glucose levels at admission, thereby overlooking the potential predictive significance of glucose variability during the hospital stay. Glycaemic variability (GV) is described as fluctuations in blood glucose levels, which result from several factors including stress-induced glucose elevation, advanced age, and medication such as insulin, adrenaline, and corticosteroids [10, 11]. Recent studies have shown that SHR and GV are potential predictors for ICU mortality in patients with IS, respectively [12, 13]. However, no study has assessed the joint effect of SHR and GV on ICU mortality in IS patients. And the emerging machine learning (ML) has shown advancement in predicting stroke-related outcomes [14], but no ML model has yet considered SHR or GV.

Therefore, the aims of this study were: (i) assessing the predictive value of SHR, GV, and their combined effects for short-term, medium-term and long-term ICU all-cause mortality in IS patients; and (ii) exploring the feasibility of SHR, GV in ML models for predicting short-term ICU all-cause mortality in IS patients (given the priority of short-term mortality prediction within the ICU).

## Methods

### Origin source of data

The data used in this analysis were extracted from the Medical Information Mart for Intensive Care version IV (MIMIC IV, version 2.0) [15]. It encompasses more than 50,000 anonymized patient records from the ICUs of the Beth Israel Deaconess Medical Center (BIDMC) during 2008 to 2019, Boston, Massachusetts, United States. The BIDMC’s Institutional Review Board has authorized the use of this database without obtaining individual consent and approved the sharing plan. One of authors (YC) was granted permission to access this database (certificate number:53753450), and has extensive experience related to the MIMIC database [16–19]. All procedures conducted in this study involving human participants were following the ethical standards of the institutional and national research committee and with the 1964 Helsinki declaration and its later amendments or comparable ethical standards.

### Study population

We first screened the ICU patients diagnosed with ischaemic stroke, as identified by specific International Classification of Diseases (ICD) version 9 codes (346.xx, 433.xx-434.xx, and 437.xx) and ICD version 10 codes (G436.x, I63.x, I65, I66, I672, I69392). The following exclusion criteria were: (i) under 18 years of age; (ii) ICU length of stay more than 24 h; (iii) measurements of blood glucose less than three times; (iv) missing data on hemoglobin A1c (HbA1c) and blood glucose records on the first day after admission; (v) patients who with multiple hospital or ICU admissions. The detailed flowchart of this study is presented in Supplementary Fig. S1.

### Extracted covariates

We extracted: (i) demographic characteristics, e.g. age, sex; (ii) vital signs records, e.g. heart rate, systolic blood pressure; (iii) the severity scores including the sequential organ failure assessment (SOFA) and the Simplified Acute Physiology Score II (SAPS II); (iv) comorbidities, e.g. hypertension, diabetes mellitus; (v) laboratory measurements, e.g. white blood cell count, platelet count; (vi) treatments, e.g. renal replacement therapy, the use of vasopressors. The details are shown in Table 1.

**Table 1:** Baseline characteristics of the 30-day survival and non-survival ICU patients with ischaemic stroke

	Total *N* = 749	Survival *N* = 575	Non-survival *N* = 174	*P*-value
**Demographic information**				
Age, years	72.9 (61.1, 83.0)	70.0 (58.8, 80.6)	81.0 (70.2, 87.6)	< 0.001
Male, n (%)	354 (47.3)	290 (50.4)	64 (36.8)	0.002
Body mass index, kg/m^2^	27.3 (23.8, 31.7)	27.7 (24.1, 31.9)	26.1 (22.8, 30.4)	0.007
Race, n (%)				0.787
White	417 (55.7)	324 (56.3)	93 (53.4)	
Black	84 (11.2)	63 (11.0)	21 (12.1)	
Other/Unknow	248 (33.1)	188 (32.7)	60 (34.5)	
**Vital signs**				
Systolic blood pressure, mmHg	143.5 ± 26.1	144.6 ± 25.3	139.9 ± 28.0	0.039
Diastolic blood pressure, mmHg	77.0 (66.0, 90.0)	77.0 (67.0, 90.0)	76.0 (61.0, 88.0)	0.115
Heart rate, bpm	81.0 (70.0, 94.0)	80.0 (69.0, 93.0)	82.5 (73.0, 97.3)	0.023
SPO_2_, %	98.0 (96.0, 100.0)	98.0 (96.0, 100.0)	99.0 (96.0, 100.0)	0.019
**Severity scores**				
SOFA	4.0 (2.0, 5.0)	3.0 (2.0, 5.0)	5.0 (3.8, 7.0)	< 0.001
SAPS II	33.0 (26.0, 40.0)	31.0 (24.0, 37.0)	39.5 (32.8, 45.0)	< 0.001
**Comorbidities**,** n (%)**				
Hypertension	403 (53.8)	308 (53.6)	95 (54.6)	0.862
Diabetes mellitus	256 (34.2)	193 (33.6)	63 (36.2)	0.524
Myocardial infarction	141 (18.8)	105 (18.3)	36 (20.7)	0.507
Peripheral vascular disease	87 (11.6)	62 (10.8)	25 (14.4)	0.224
Congestive heart failure	214 (28.6)	153 (26.6)	61 (35.1)	0.035
Chronic kidney disease	136 (18.2)	96 (16.7)	40 (23.0)	0.072
Chronic pulmonary disease	126 (16.8)	97 (16.9)	29 (16.7)	0.950
Malignant cancer	51 (6.8)	31 (5.4)	20 (11.5)	0.009
Metastatic solid tumor	23 (3.1)	13 (2.3)	10 (5.7)	0.040
Liver disease	26 (3.5)	19 (3.3)	7 (4.0)	0.639
**Treatments**,** n (%)**				
Renal replacement therapy	14 (1.9)	9 (1.6)	5 (2.9)	0.334
Mechanical ventilation	236 (31.5)	145 (25.2)	91 (52.3)	< 0.001
Vasopressor	127 (17.0)	82 (14.3)	45 (25.9)	0.001
Antiplatelet agent	307 (41.0)	237 (41.2)	70 (40.2)	0.861
Anticogulant	28 (3.7)	25 (4.3)	3 (1.7)	0.168
Statin	278 (37.1)	214 (37.2)	64 (36.8)	0.929
Insulin	538 (71.8)	401 (69.7)	137 (78.7)	0.021
**Laboratory measurements**				
Serum creatinine, mg/dL	0.9 (0.7, 1.2)	0.9 (0.7, 1.1)	1.0 (0.8, 1.4)	0.001
eGFR, mL/min/1.73m^2^	76.4 (55.5, 100.5)	79.9 (58.8, 101.7)	65.0 (44.6, 86.1)	< 0.001
White blood cells, 10^9^/L	10.2 (7.9, 13.1)	10.1 (7.8, 12.9)	11.0 (8.2, 13.8)	0.031
Hemoglobin, g/dL	12.3 (10.9, 13.7)	12.5 (11.1, 13.8)	11.4 (9.8, 12.8)	< 0.001
Platelets, 10^9^/L	215.0 (171.5, 270.5)	212.0 (173.0, 270.0)	218.5 (166.0, 272.0)	0.972
Glucose, mg/dL	123.0 (102.0, 158.5)	120.0 (100.0, 156.0)	132.0 (107.8, 168.0)	0.006
Calcium, mEq/L	8.7 (8.2, 9.1)	8.8 (8.3, 9.1)	8.4 (8.0, 8.9)	< 0.001
Sodium, mEq/L	139.0 (137.0, 142.0)	139.0 (137.0, 142.0)	140.0 (137.0, 143.0)	0.068
Potassium, mEq/L	4.1 (3.7, 4.4)	4.0 (3.7, 4.4)	4.2 (3.8, 4.6)	0.029
HbA1c, %	5.8 (5.4, 6.6)	5.8 (5.4, 6.6)	5.9 (5.4, 6.6)	0.799
**Indicators of interest**				
SHR	1.0 (0.9, 1.2)	1.0 (0.9, 1.2)	1.1 (0.9, 1.3)	< 0.001
GV, %	15.9 (10.6, 22.4)	15.0 (10.1, 21.1)	20.1 (12.1, 29.2)	< 0.001

Abbreviations: *eGFR* estimated glomerular filtration rate, *GV* glycaemic variability, *HbA1c* hemoglobin A1c, *ICU* intensive care unit, *SAPS II* Simplified Acute Physiology Score II, *SHR* stress hyperglycaemic ratio, *SOFA* Sequential Organ Failure Assessment, *SPO*_*2*_ saturation of peripheral oxygen

### Definitions of SHR and GV

SHR was defined as the index calculated using the following equation: SHR = (first blood glucose within first day after admission/[28.7 × first HbA1c within first day after admission − 46.7]), the units of blood glucose and HbA1c were mg/dL and %, respectively [6]. GV was quantified by coefficient of variation, which calculates the standard deviation relative to the average of all the repeated glucose assessments [20].

### Study outcomes

The primary outcome of the present study was 30-day ICU all-cause mortality. The secondary outcomes were 90-day, 360-day ICU all-cause mortality. Other outcomes including ICU all-cause mortality, hospital all-cause mortality, ICU length of stay, and hospital length of stay were only described briefly.

### Statistical analysis

The initially extracted data had varying proportions of missing values (Supplementary Table S1), and we performed multiple interpolation in Python. Continuous variables of normal distribution were represented as mean with standard deviation or median with interval quartile range (IQR), and analysed using Student t-test or analysis of variance. The analysis of non-normally distributed variables was conducted using the Mann-Whitney U test or Kruskal–Wallis test. Categorical variables were presented as numbers and percentages (%) and analysed using Chi-square test or Fisher exact test.

The SHR and GV distributions were classified into tertiles as follows: SHR (tertile 1 [T1]: SHR ≤ 0.90; T2: 0.90 < SHR ≤ 1.12; T3: SHR > 1.12) and GV (T1: GV ≤ 12.20%; T2: 12.20% < GV ≤ 20.14%; T3: GV > 20.14%). The T3 was categorized as high group, while the remaining T1 and T2 were classified as low group. In investigating the combined effect of SHR and GV, we defined four groups, respectively, G1: Low SHR and low GV; G2: High SHR and low GV; G3: Low SHR and high GV; G4: High SHR and high GV. Using Kaplan-Meier curves, the probability of 360-day survival was compared for combinations of different groups of SHR and GV in the overall and subgroups (DM, non-DM), and differences were assessed using the log-rank test. Furthermore, potential nonlinear relationships between SHR, GV, and all-cause mortality were examined by using restricted cubic spline (RCS) analysis in the overall and subgroups (DM, non-DM).

Then, to investigate the correlations between SHR, GV, and their combined effect on mortality, logistic regression and Cox proportional hazards regression analyses were conducted. All Cox regression analyses satisfied the proportional hazards assumption, as detailed in Supplementary Table S20. Initially, univariate assessments were performed for each potential variable. Only variables that showed significance in the univariate analysis, as well as those considered clinically relevant, were included in the subsequent adjusted multivariate analysis. Therefore, we adjusted for several models, Model 1: Unadjusted; Model 2: Adjusted for age, sex, race, body mass index (BMI), systolic blood pressure, diastolic blood pressure, heart rate, SAPS II, SOFA, saturation of peripheral oxygen; Model 3: Model2 further adjusted hypertension, DM, myocardial infarction, peripheral vascular disease, congestive heart failure, chronic kidney disease, chronic pulmonary disease, liver disease, metastatic solid tumour, malignant cancer, renal replacement therapy, the use of mechanical ventilation (MV), the use of vasopressor, antiplatelet agent, anticoagulant, statin, insulin, eGFR, white blood cell, haemoglobin, platelet, calcium, sodium, potassium. Subsequently, subgroup analyses were used to explore whether the associations between SHR or GV or the combination of SHR with GV and outcomes were stable across subgroups (focus: DM vs. non-DM; others: age < 70 years vs. Age ≥ 70 years; male vs. female; BMI < 30 kg/m^2^ vs. BMI ≥ 30 kg/m^2^), and interactions with each subgroup variable were calculated.

The predictive value of SHR, GV, and their combined effects on 30-day mortality, 90-day mortality and 360-day mortality were assessed using receiver operating characteristic (ROC) curves. Hanley & McNeil’s method was employed to compare the predictive performances of SHR, GV, and their combination.

Due to the urgency of predicting short-term deaths in critically ill IS patients, and to demonstrate the importance of SHR and GV in the ML prediction of 30-day all-cause ICU mortality in critically ill IS patients, we also construct a ML prediction model. First, we divide the whole cohort into *training cohort* and *internal validation cohort* by 7:3. Second, Boruta was used in the training cohort to pre-screen the features that were important for our target outcome. Third, Pearson’s correlation and variance inflation factor test were performed on the selected features to avoid covariance and multicollinearity, if without covariance and multicollinearity among features, then the pre-selected features were import into six common ML algorithms for medical binary classification tasks, including light gradient boosting machine (LightGBM), random forest, logistic regression, Support Vector Machine, k-nearest neighbours, and multilayer perceptron. Fourth, we plotted the ROC and area under curve (AUC) with 95% CI of each ML model and assessed their performance based on accuracy, specificity, precision, recall, F1-score, and G-mean, and compared with the SOFA, a commonly used mortality score in ICUs. Fifth, for the selected best model, we used SHapley Additive exPlanations (SHAP) to visualise the contribution of the features in the model to the target outcome, and generated the partial dependence plot of a feature in the model to explain the relationship between this feature and the target outcome. Sixth, to assess the incremental predictive value of adding SHR and GV, we calculated the net reclassification improvement (NRI) and integrated discrimination improvement (IDI) compared with a baseline best-performance model including all variables within the best-performing model except SHR and GV. Both indices were estimated using 1,000 bootstrap replications to obtain bias-corrected 95% CIs. Finally, we additionally constructed an easy-to-use online prediction tool based on the selected model to enhance the clinical usability.

In this study, we conducted the following sensitivity analyses: (i) To adequately reflect stress-induced hyperglycemia throughout the entire ICU stay, we further explored the relationship between GV and SHR within the first 48 h of ICU admission and their association with all-cause mortality. (ii) To strengthen the rationale for using the GV as an indicator of blood glucose fluctuation, we performed a sensitivity check by using standard deviation (SD) as an alternative measure to verify the stability of the results. (iii) We further divided SHR and GV into quartiles to explore their relationship with ICU all-cause mortality, providing more detailed insights. (iv) We excluded patients with very few blood glucose measurements (*n* < 3) for sensitivity testing, to avoid potentially unreliable estimates of GV.

Statistical analyses were conducted using SPSS software (version 26.0, USA), R (version 4.3.1, Austria), and Python (3.11.1, USA) The value of two-tailed *P* < 0.05 was considered to be statistically significant in this study.

## Results

### Baseline characteristics

A total of 749 patients were enrolled in this study, the median with IQR of age was 72.9 (61.1, 83.0) years, and 354 (47.3%) were males. For the study outcomes, the 30-day, 90-day, and 360-day ICU mortality were 23.2%, 29.6%, and 35.4%, respectively; the ICU and hospital mortality were 10.4% and 16.2, respectively; and the length of stay in the ICU and the length of hospital stay were 4.4 (2.9, 4.6) days and 8.7 (5.2, 14.7) days, respectively. Compared with the patients of 30-day ICU survival group, the patients of 30-day ICU non-survival group were older, had higher proportion of females, higher SOFA and SAPSII, and had more congestive heart failure and malignancy (details are given in Table 1).

### Associations of SHR and 30-day, 90-day, 360-day ICU all-cause mortality

**Fig. 1:**
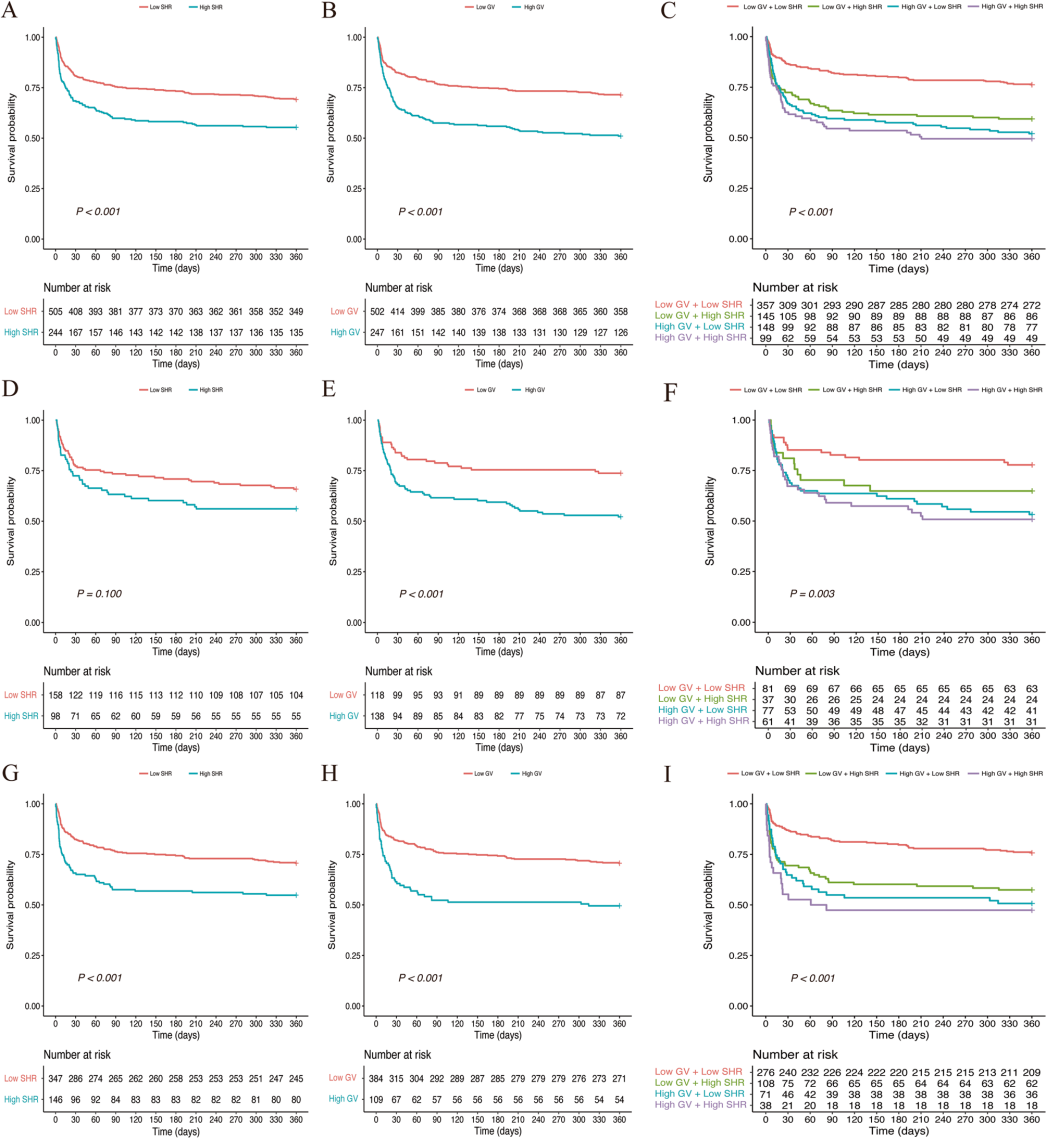
Kaplan-Meier curves of SHR, GV, and their combination for 360-day mortality. (**A**-**C**) Overall population; (**D**-**F**) patients with diabetes; (**G**-**I**) patients without diabetes. Abbreviations: GV, glycemic variability; SHR, stress hyperglycemia ratio

Kaplan-Meier curves showed that in both the overall population (Fig. 1A) and non-DM population (Fig. 1D), the cumulative 360-day ICU all-cause mortality rates were significantly higher in the high SHR group than in the low SHR group (both log-rank *P* < 0.001), whereas in the DM population [Fig. 1G] there was no significant difference between the two groups (*P* = 0.100). However, the ICU all-cause mortality rates at 30-day, 90-day, and 360-day were higher in the high SHR group than in the low SHR group, regardless of whether DM was coexistent or not. The results of the RCS analyses in Supplementary Fig. S2 suggest that SHR is linearly associated with 30-day, 90-day and 360-day ICU all-cause mortality in the overall population or non-DM population, but SHR is only linearly associated with 360-day all-cause mortality in the DM population.

Since T1 of SHR had the lowest 30-day mortality rate (18.1%), and therefore, T1 was used as the reference group in the Cox proportional hazards models analysis (Supplementary Table S2). According to Supplementary Table S3, Supplementary Table S4, and Supplementary Table S5: (i) In the overall population, high levels of SHR were significantly associated with 30-day, 90-day, and 360-day ICU all-cause mortality (for 30-day: T1 as the reference group, T3 HR 1.65 95% CI 1.09–2.49; for 90-day: T1 as the reference group, T3 HR 1.62 95% CI 1.13–2.33; for 360-day: T1 as the reference group, T3 HR 1.50 95% CI 1.08–2.10). (ii) In the non-DM population, high levels of SHR were significantly associated with 30-day, 90-day, and 360-day ICU all-cause mortality (for 30-day: T1 as the reference group, T2 HR 2.00 95% CI 1.14–3.50, T3 HR 2.93 95% CI 1.65–5.19; for 90-day: T1 as the reference group, T3 HR 2.40 95% CI 1.47–3.91; for 360-day: T1 as the reference group, T3 HR 2.16 95% CI 1.38–3.38). (iii) In the DM population, SHR were not associated with 30-day, 90-day, and 360-day ICU all-cause mortality. Moreover, no significant interaction was found for the results of other subgroup analyses (Supplementary Table S6, Supplementary Table S7, and Supplementary Table S8). Moreover, the AUCs of SHR alone for predicting 30-day, 90-day, and 360-day ICU all-cause mortality were 0.588, 0.591, and 0.570, respectively (Supplementary Fig. S3).

### Associations of GV and 30-day, 90-day, 360-day ICU all-cause mortality

Kaplan-Meier curves showed that in the overall, DM and non-DM populations (Fig. 1B and E, and Fig. 1H), the cumulative 360-day ICU all-cause mortality rates were significantly higher in the high GV group than in the low GV group (all log-rank *P* < 0.001). Moreover, the ICU all-cause mortality rates at 30-day and 90-day were also higher in the high GV group than in the low GV group. The results of the RCS analyses in Supplementary Fig. S4 show that GV is associated with 30-day, 90-day and 360-day ICU all-cause mortality, regardless of the presence or absence of DM.

Since T2 of GV had the lowest 30-day mortality rate (17.3%), and therefore, T2 was used as the reference group in the Cox proportional hazards models analysis (Supplementary Table S9). According to Supplementary Table S3, Supplementary Table S4, and Supplementary Table S5: (i) In the overall population, high levels of GV were significantly associated with 30-day, 90-day, and 360-day ICU all-cause mortality (for 30-day: T2 as the reference group, T1 HR 1.63 95% CI 1.05–2.53, T3 HR 2.18 95% CI 1.44–3.31; for 90-day: T2 as the reference group, T3 HR 1.75 95% CI 1.22–2.50; for 360-day: T2 as the reference group, T3 HR 1.51 95% CI 1.09–2.09). (ii) In the non-DM population, high levels of GV were significantly associated with 30-day, 90-day, and 360-day ICU all-cause mortality (for 30-day: T2 as the reference group, T1 HR 1.96 95% CI 1.16–3.29, T3 HR 2.80 95% CI 1.66–4.73; for 90-day: T2 as the reference group, T3 HR 2.15 95% CI 1.37–3.38; for 360-day: T2 as the reference group, T3 HR 1.80 95% CI 1.18–2.73). (iii) In the DM population, GV were not associated with 30-day, 90-day, and 360-day ICU all-cause mortality. Moreover, except for 30-day ICU all-cause mortality, there was a significant interaction between GV and BMI < 30 kg/m^2^, no significant interaction was found for the results of other subgroup analyses (Supplementary Table S10, Supplementary Table S11, and Supplementary Table S12). Furthermore, the AUCs of GV alone for predicting 30-day, 90-day, and 360-day ICU all-cause mortality were 0.619, 0.624, and 0.622, respectively (Supplementary Fig. S3).

### Associations of SHR combined with GV and 30-day, 90-day, 360-day ICU all-cause mortality

Kaplan-Meier curves showed that in the overall, DM and non-DM populations (Fig. 1C and F, and Fig. 1I), high SHR with high GV group had the highest cumulative 360-day all-cause mortality, followed in descending order by the high GV with low SHR group, the low GV with high SHR group, and the low GV with low SHR group (all log-rank *P* < 0.01). And, there were similar trends in all-cause mortality at 30-day and 90-day.

**Fig. 2:**
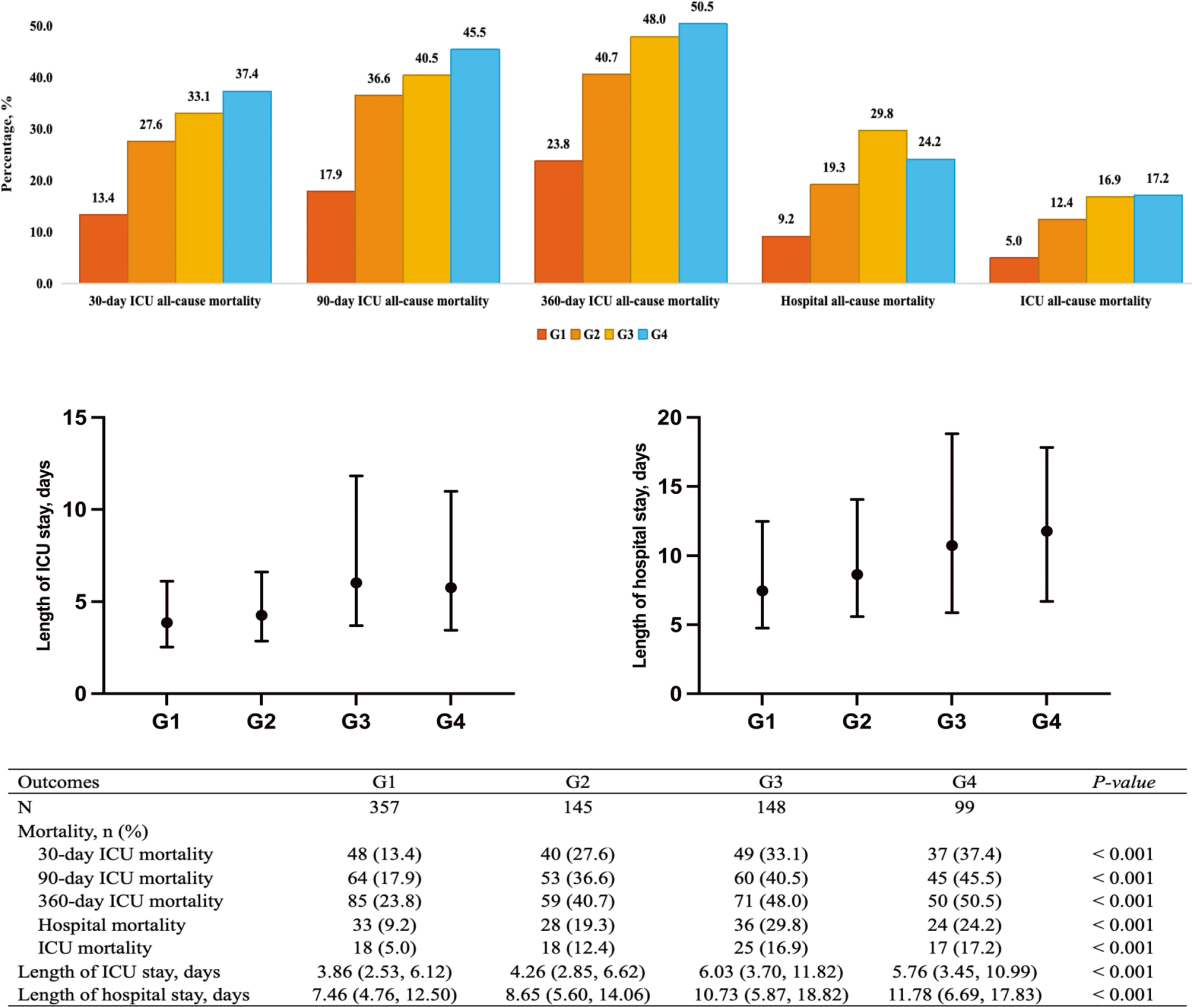
Outcomes of ischaemic stroke patients according to groups of SHR combine with GV. G1: Low SHR and low GV (SHR ≤ 1.12 and GV ≤ 20.14%); G2: High SHR and low GV (SHR > 1.12 and GV ≤ 20.14%); G3: Low SHR and high GV (SHR ≤ 1.12 and GV > 20.14%); G4: High SHR and high GV (SHR > 1.12 and GV > 20.14%). ICU, intensive care unit; GV, glycaemic variability; SHR, stress hyperglycaemic ratio

Since G1 of GV had the lowest 30-day mortality rate (13.4%), and therefore, G1 was used as the reference group in the Cox proportional hazards models analysis (Fig. 2). According to Table 2: (i) In the overall population, G2, G3, G4 were significantly associated with higher risk of 30-day, 90-day, and 360-day ICU all-cause mortality compared with G1 (for 30-day: G1 as the reference group, G2 HR 1.83 95% CI 1.16–2.86, G3 HR 2.22 95% CI 1.42–3.48, G4 HR 2.43 95% CI 1.42–4.14; for 90-day: G1 as the reference group, G2 HR 1.94 95% CI 1.31–2.86, G3 HR 2.06 95% CI 1.39–3.05, G4 HR 2.18 95% CI 1.36–3.06; for 360-day: G1 as the reference group, G2 HR 1.76 95% CI 1.23–2.51, G3 HR 1.75 95% CI 1.23–2.49, G4 HR 1.77 95% CI 1.14–2.74). (ii) In the non-DM population, G2, G3, G4 were significantly associated with increased risk of 30-day, 90-day, and 360-day ICU all-cause mortality compared with G1 (for 30-day: G1 as the reference group, G2 HR 2.11 95% CI 1.25–3.55, G3 HR 2.28 95% CI 1.27–4.08, G4 HR 4.10 95% CI 1.27–8.35; for 90-day: G1 as the reference group, G2 HR 2.02 95% CI 1.28–3.17, G3 HR 2.01 95% CI 1.20–3.37, G4 HR 3.29 95% CI 1.74–6.21; for 360-day: G1 as the reference group, G2 HR 1.82 95% CI 1.20–2.75, G3 HR 1.64 95% CI 1.02–2.65, G4 HR 2.76 95% CI 1.50–5.07). (iii) In the DM population, combination of SHR and GV was not associated with 30-day, 90-day, and 360-day ICU all-cause mortality. Additionally, the AUCs of combination of SHR and GV for predicting 30-day, 90-day, and 360-day ICU all-cause mortality were 0.643, 0.652, and 0.640, respectively (Supplementary Fig. S3). The combination of SHR and GV was significantly superior to either SHR or GV alone when predicting 30-day and 90-day ICU all-cause mortality (Hanley & McNell *P* < 0.05), but only significantly superior to SHR alone when predicting 360-day ICU all-cause mortality (Hanley & McNell *P* < 0.05).

**Table 2:** The relationship of the combination of SHR and GV with mortality in ischaemic stroke patients

	30-day ICU mortality		90-day ICU mortality		360-day ICU mortality	
	HR (95% CI)	*P-value*	HR (95% CI)	*P-value*	HR (95% CI)	*P-value*
**Overall**						
G1	*Reference*		*Reference*		*Reference*	
G2	1.83 (1.16, 2.86)	0.009	1.94 (1.31, 2.86)	0.001	1.76 (1.23, 2.51)	0.002
G3	2.22 (1.42, 3.48)	< 0.001	2.06 (1.39, 3.05)	< 0.001	1.75 (1.23, 2.49)	0.002
G4	2.43 (1.42, 4.14)	0.001	2.18 (1.36, 3.06)	< 0.001	1.77 (1.14, 2.74)	0.011
**Patients without diabetes mellitus**						
G1	*Reference*		*Reference*		*Reference*	
G2	2.11 (1.25, 3.55)	0.005	2.02 (1.28, 3.17)	0.002	1.82 (1.20, 2.75)	0.005
G3	2.28 (1.27, 4.08)	0.006	2.01 (1.20, 3.37)	0.008	1.64 (1.02, 2.65)	0.042
G4	4.10 (1.27, 8.35)	< 0.001	3.29 (1.74, 6.21)	< 0.001	2.76 (1.50, 5.07)	0.001
**Patients with diabetes mellitus**						
G1	*Reference*		*Reference*		*Reference*	
G2	1.90 (0.61, 5.85)	0.266	2.30 (0.92, 5.80)	0.076	2.15 (0.94, 4.89)	0.069
G3	1.99 (0.85, 4.64)	0.112	1.93 (0.92, 4.03)	0.082	1.79 (0.93, 3.43)	0.081
G4	1.64 (0.61, 4.39)	0.326	1.68 (0.71, 3.95)	0.235	1.39 (0.64, 3.02)	0.405

G1: Low SHR and low GV (SHR ≤ 1.12 and GV ≤ 20.14%); G2: High SHR and low GV (SHR > 1.12 and GV ≤ 20.14%); G3: Low SHR and high GV (SHR ≤ 1.12 and GV > 20.14%); G4: High SHR and high GV (SHR > 1.12 and GV > 20.14%)

Model: Adjusted for age, sex, race, body mass index, systolic blood pressure, diastolic blood pressure, heart rate, saturation of peripheral oxygen, Simplified Acute Physiology Score II, Sequential Organ Failure Assessment, hypertension, diabetes mellitus, myocardial infarction, peripheral vascular disease, congestive heart failure, chronic kidney disease, chronic pulmonary disease, liver disease, metastatic solid tumor, malignant cancer, renal replacement therapy, mechanical ventilation, vasopressor, antiplatelet agent, anticogulant, statin, insulin, estimated glomerular filtration rate, white blood cell, hemoglobin, platelet, calcium, sodium, potassium

Abbreviations: *CI* confidence interval, *GV* glycaemic variability, *HR* hazard ratio, *ICU* intensive care unit, *SHR* stress hyperglycaemic ratio

### Feature selection for the ML models

After dividing the training cohort and internal validation cohort according to 7:3 and performing Boruta screening in the training cohort (Supplementary Fig. S5), a total of seven significant features for 30-day ICU all-cause mortality were obtained, of which GV and SHR were ranked third and fourth, respectively, and the rest of the features included MV, age, eGFR, SpO_2_, hemogobin. Supplementary Fig. S6 shows that there is no significant correlation or multicollinearity for these seven variables.

### ML models construction and evaluation

**Fig. 3:**
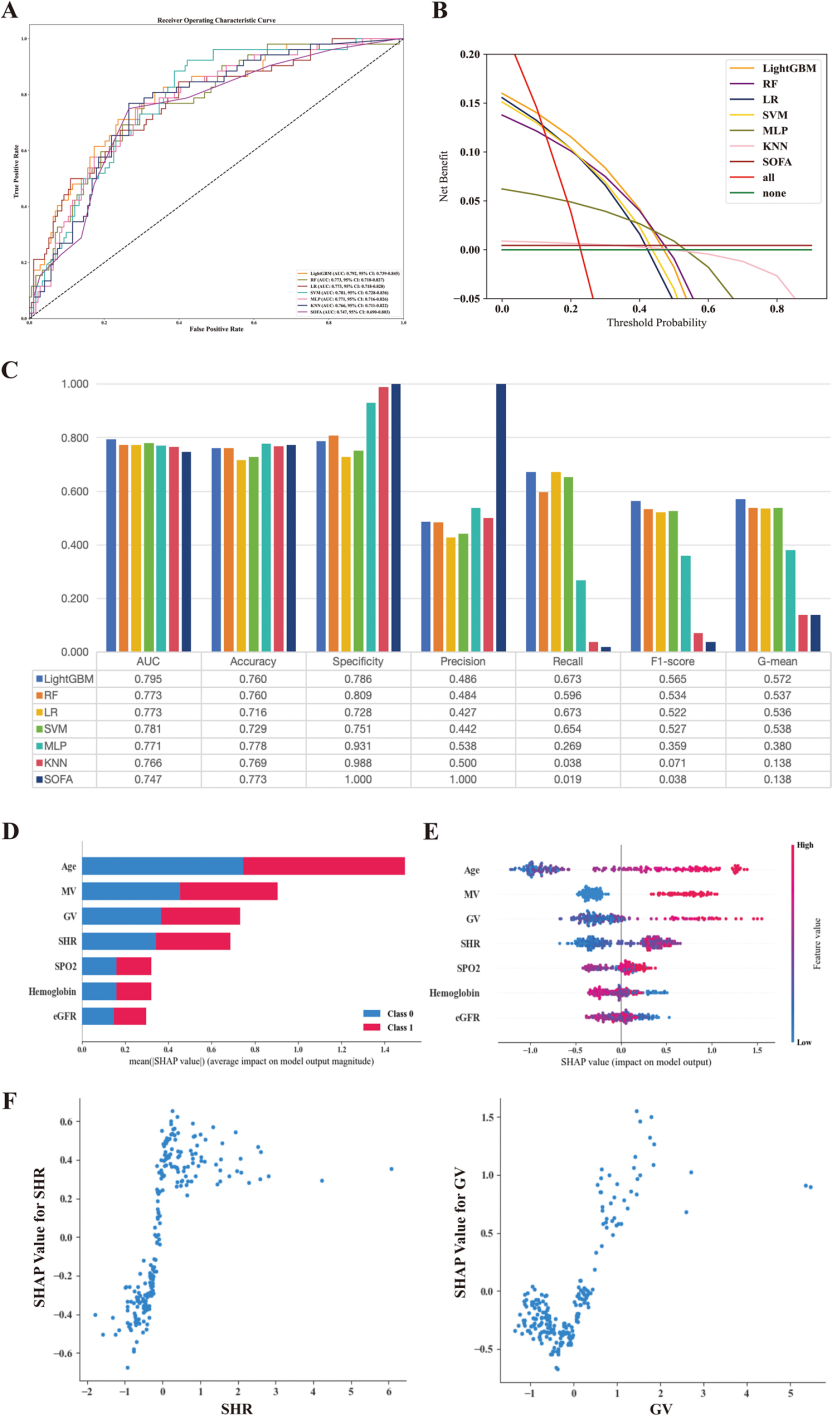
Evaluation the performance of machine learning models in the *internal validation cohort*, and SHAP plots of features within the best model. ROC curves (**A**), decision curve analysi (**B**), other metrics (**C**), feature importance of the best model (**D**, **E**), and dependence plots of SHR and GV (**F**). AUC, area under curve; eGFR, estimated glomerular filtration rate; GV, glycaemic variability; KNN, k-nearest neighbors; LightGBM, light gradient boosting machine; LR, logistic regression; MLP, multilayer perceptron; MV, mechanical ventilation; RF, random forest; SHAP, SHapley Additive exPlanations; SHR, stress hyperglycaemic ratio; SOFA, sequential organ failure assessment; SVM, Support Vector Machine

These seven features were input into six ML models, and the optimal hyperparameters for each ML model were determined through 5-fold cross-validation and manual fine-tuning (Supplementary Table S13). Then, the performances of all ML models were evaluated in the *internal validation set*. Figure 3A and B, and Fig. 3C show the ROC curves, DCA curves, AUCs and 95% CIs, and several other metrics for all ML models, all of which outperformed traditional SOFA. Among these ML models, LightGBM was considered the best ML model because it had the highest AUC (0.795), F1-score (0.565), and G-mean (0.572), and had the largest net clinical benefit.

### Visualisation of feature importance

Based on the LightGBM model, we calculated the SHAP values of different features. Figure 3D and E show the order of importance obtained according to the SHAP value of each feature, with GV and SHR remaining in the third and fourth positions. And the partial dependence plots showed that increased SHR and GV were associated with 30-day ICU all-cause mortality (Fig. 3F).

### Incremental predictive value of SHR and GV in the ML model

The inclusion of SHR and GV significantly improved discrimination over the baseline LightGBM model excluding these variables. The NRI was 14.6% (95% CI: 5.8%–23.0%) and IDI was 10.9% (95% CI: 8.3%–13.4%) in the training cohort, with consistent results in the testing cohort (NRI: 7.9%, 95% CI: 2.0%–13.1%; IDI: 6.7%, 95% CI: 2.3%–11.1%).

### Web-based prediction platform

To overcome the lack of feasibility of external and clinical applications of ML model, we embedded the LightGBM model into a simple and easy-to-use web-based prediction platform, which can be easily validated or predicted by external users and clinicians. The detailed web address is “http://43.165.2.195/”. For example, in Supplementary Fig. S7, a patient, 70 years, whose GV is 15.5%, SHR is 1.27, eGFR is 43.5mL/min/1.73m^2^, SpO2 is 98%, haemoglobin is 9.3 g/dL, and who is also receiving MV. The final output probability for 30-day ICU all-cause mortality is 0.73, suggesting this patient is at high-risk.

### Sensitivity analysis

Supplementary Tables 14 and 15 present sensitivity analyses of GV and SHR calculated from blood glucose levels within 48 h of ICU admission against mortality. Results indicate that both SHR and GV were significantly associated with all-cause mortality in the ICU, both in the overall cohort and in non-diabetic patients, whereas no significant association was observed in diabetic patients. Higher glycaemic classifications (G2, G3, G4) were also associated with increased mortality risk in both the overall cohort and non-diabetic patients. Supplementary Table S16 demonstrates the relationship between SD and various mortality rates. Results indicate that in the overall population and non-diabetic cohort, SD is significantly associated with 30/90/360-day all-cause mortality in the ICU. Conversely, within the diabetic cohort, SD is significantly associated with long-term ICU mortality (90/360-day). Further sensitivity analysis was conducted after dividing SHR and GV into quartiles. Supplementary Table 17 demonstrates that both SHR and GV were significantly associated with all-cause mortality in the ICU, regardless of whether the population was general or non-diabetic. However, no significant association was observed in the diabetic population. Moreover, the risk of harm increased with progressively higher quartiles of SHR and GV. After excluding patients with fewer than three glucose measurements during ICU stay, Supplementary Tables 18 and 19 demonstrate that both SHR and GV were significantly associated with 30-day, 90-day, and 360-day all-cause mortality in the overall population and non-diabetic cohort. The risk effect intensified with increasing SHR and GV levels, whereas no significant association was observed in the diabetic cohort. Higher glycaemic classifications (G2, G3, G4) were also associated with increased mortality risk in both the overall population and non-diabetic cohort.

## Discussion

In this study, we assessed the combined effect of SHR and GV on all-cause mortality in ICU-admitted IS patients. Our research yielded several findings that hold clinical significance and warrant attention: (i) Linear relationships were observed between SHR or GV with 30-day, 90-day, 360-day ICU all-cause mortality. (ii) Both SHR and GV showed significant associations with mortality in non-DM individuals; however, these associations weakened and became non-significant within DM individuals. (iii) Integration of SHR and GV was more effective than SHR or GV alone in predicting mortality. (iv) We constructed a well-performing ML model using SHR, GV, and other clinical parameters, noting that SHR and GV were in the forefront of importance to the ML model. Overall, combined SHR and GV have an important role in risk stratification of overall IS critically ill patients.

Glucose metabolism is associated with a wide range of prognoses in IS patients. Post-stroke hyperglycaemia is present in all patients with a history of diabetes, accounting for approximately 37% of IS patients and 50% of IS patients without DM [21]. In individuals without a documented history of diabetes, the occurrence of acute hyperglycaemia is attributed to a systemic stress reaction and elevated glucocorticoid levels. In the ICU set, IS patients are at a higher risk of dramatic blood sugar fluctuations and stress-induced hyperglycemia due to the dual effects of stress-induced insulin resistance and sympathetic nervous system activation. Research indicates that hyperglycemia lead to inflammatory reactions and oxidative stress, compromising blood-brain barrier integrity, thereby increasing infarct size, brain edema, hemorrhagic transformation, neuronal loss, and neurological deficits, ultimately raising the risk of mortality [22, 23]. Moreover, in IS patients receiving intravenous tissue plasminogen activator (tPA) therapy, hyperglycemia has been linked to reduced rates of recanalization and an increased risk of hemorrhagic transformation, further heightening the chances of mortality [24–28]. Potential pathological factors contributing to inadequate tPA recanalization in stroke patients include elevated levels of plasminogen activator inhibitor 1 (PAI-1), atherosclerotic vascular damage due to diabetes, glycation of the tPA receptor annexin A2, and alterations in fibrin clot density [29, 30].

Acute stress hyperglycemia, a neuroendocrine system response involving the sympathetic nervous system and hypothalamic-pituitary-adrenal axis, has been linked to increased mortality rates and other adverse endpoints [31, 32]. Numerous studies have identified the SHR as a predictor of heightened risk for adverse events and death in individuals with IS [8, 9, 33–39]. Importantly, the effects of stress-induced hyperglycemia vary by DM status. Severe stress hyperglycemia in non-DM patients raises mortality risks, whereas patients with DM may not show the same pattern possibly due to chronic exposure to elevated glucose levels [37, 40, 41]. This observation is consistent with the findings of the current study. Additionally, interventions to lower glucose levels during hospitalization can alter blood sugar levels, impacting the patient’s prognosis. Therefore, relying solely on the initial admission glucose value to calculate the SHR may not accurately reflect the influence of glucose fluctuations during hospitalization on the overall prognosis.

High GV is also an indication of disrupted glucose homeostasis. In IS patients, heightened GV has been associated with unfavorable functional prognosis [42], early neurological decline [43], diminished cognitive performance [44], increased risk of HT [45], and a higher incidence of major adverse cardiovascular events [46]. These consequences collectively contribute to an elevated likelihood of early mortality among such patients. Base on this perspective, it could be hypothesized that heightened GV may not simply act as an indicator of disease severity, but rather play an active role in the progression of stroke deterioration. High GV can result in the generation of oxidative stress, leading to an increase in intracellular reactive oxygen species. Consequently, this may lead to various consequences such as impaired angiogenesis in response to ischaemic conditions, the activation of inflammatory pathways, and ultimately the apoptosis of endothelial cells [47].Furthermore, it induces lasting epigenetic changes that perpetuate the sustained activation of proinflammatory genes even after normal glycemic levels have been restored, which is called the “metabolic memory phenomenon” [6]. These mechanisms might explain the poor long-term outcomes of high GV, whereas the poor short-term outcomes such as infarct size expansion and HT could be mediated by hyperglycemia. Our study identified a significant association between GV and mortality in the overall population of IS patients. However, in the subgroup analysis, this association was observed only in non-diabetes patients. The finding was consistent with the subgroup analysis of SHR in this study. In addition to chronic exposure to hyperglycemia, patients with DM may also exhibit increased tolerance to lower or higher levels of blood glucose [48].

This study represents the first to combine the SHR and GV in assessing mortality risk among critically ill patients with IS. Our findings indicate that the combined assessment of SHR and GV significantly enhances predictive accuracy for 30-day, 90-day, and 360-day mortality. Compared to using SHR or GV alone, this integrated approach demonstrates superior predictive value in clinical settings. Previous studies have extensively examined SHR and GV as independent predictors of mortality risk. SHR, as an indicator of stress-induced hyperglycaemia, has been confirmed by multiple investigations to correlate closely with outcomes in critically ill patients suffering from conditions such as sepsis and cardiovascular and cerebrovascular diseases [49–52], particularly in acute stroke patients where elevated SHR correlates with increased mortality risk [53, 54]. Furthermore, GV, reflecting blood glucose variability, is also recognised as a key prognostic factor for critically ill patients [55]. Recent studies, Cai et al. have demonstrated that elevated GV correlates with poor outcomes, particularly in critically ill stroke patients [54, 56]. However, despite demonstrating significant clinical relevance in independent prediction, the use of these metrics in isolation presents limitations. SHR relies solely on admission blood glucose levels, whilst GV primarily reflects intra-hospital glucose variability. Combining both provides more comprehensive prognostic information. Our study, integrating SHR and GV, reveals their potent predictive capability in critically ill stroke patients through synergistic effects. We found that the combination of high SHR and high GV (G4 group) significantly increased mortality risk at all time points (30 days, 90 days, and 360 days), with hazard ratios (HR) of 2.43, 2.18, and 1.77 respectively. This indicates that combined assessment holds greater prognostic value than using SHR or GV alone.

How does our LightGBM model of ICU 30-day all-cause mortality in IS patients compare with previous ones? Huang et al. constructed a ML model in predicting 28-day hospital all-cause mortality in older IS patients (aged ≥ 65 years), and their best model was XGBoost, which had an AUC of 0.733 [57]. Compared with our LightGBM, our ML model performed better and used only 7 features. In addition, other ML models associated with stroke-related mortality had differences in ML model performance due to some differences in the target population and target outcomes from our study [58, 59]. However, none of the previous stroke-related ML models considered features related to glucose metabolism other than glucose, and our results highlight to the necessity of considering SHR and GV in IS prognostic prediction. In addition, the poor utility of the ML in previous studies again emphasised the sophisticated predictive platform of the LightGBM model in our study.

Due to the critical role of SHR and GV in predicting prognosis, our study aimed to apply the combination of SHR and GV to improve the risk stratification of IS patients. Our study indicated that non-DM patients with both stress-induced hyperglycemia and severe blood glucose fluctuations have a poor prognosis. In critically ill patients without diabetes, the body may lose its ability to maintain glucose homeostasis, leading to heightened detrimental impacts. When these patients experience the “double blow” of stress-induced hyperglycemia and high GV, the consequences can be particularly grave. The findings of this study may hold significant implications for blood glucose management practices in ICU-admitted IS patients. For non-DM patients with severe conditions, physicians need to closely monitor their blood glucose levels, striving to prevent stress-induced hyperglycemia. Should stress-induced hyperglycemia occur, a gradual reduction in blood glucose levels is recommended to minimize glucose fluctuations. Therefore, the combined assessment of SHR and GV may aid in guiding personalized blood glucose management in the future, ultimately improving patient outcomes.

### Strengths and limitations

Our study has important strengths in assessing the combined effect of SHR and GV on poor prognosis in IS patients, as well as the use of SHR and GV in models of IS-associated ML, which have not been explored in previous studies. The assessments were comprehensive, utilizing the extensive MIMIC-IV public database and one-year follow-up data to assess both short- and long-term consequences. However, the study has some limitations. First, due to its retrospective observational design, establishing a definitive cause-and-effect relationship between exposure and outcome is complex, and retrospective bias could not be avoided. Also, focusing on all-cause mortality as an outcome may overlook contributions from other fatal causes. Second, the study included ICU-admitted IS patients who primarily receive insulin therapy for glycemic control, excluding oral antidiabetic drugs. The exclusion of lab parameters with significant data gaps could introduce bias. Third, our SHR was calculated on the basis of the first value on admission, so fluctuations in SHR were not taken into account, and patients did not have the same number of blood glucose measurements, so random errors could not be avoided. Fourth, factors like dietary intake, nutritional support, insulin dosage adjustments, and administration schedules may influence blood glucose variability. Fifth, Considering the fairly limited size of the DM population, the assessment of the combined effects of SHR and GV may be biased. Sixth, as the validation of ML also relies on relatively large sample sizes, based on the current data, we are unable to validate the predictive performance of LightGBM in DM and non-DM populations separately. We acknowledge the risk of overfitting given our modest sample size and reliance on internal validation; accordingly, we temper our claims and note that independent external validation, across centers and time periods, ideally prospective, is needed to confirm generalizability. Seventh, due to limitations in data availability within the mimic database, we did not investigate the impact of the National Institutes of Health Stroke Scale and pre-stroke disability measures on model performance, particularly in comprehending the full variability of prognostic outcomes across different patient cohorts. Future studies incorporating neurological severity scales may enhance the model’s prognostic value and clinical applicability, thereby enabling a more comprehensive assessment of outcomes for intensive care unit patients. Eighth, one limitation of our study is the inability to perform a sensitivity analysis by ICU subtype (medical, neurological, surgical) due to the small sample size in each group, which would have reduced statistical power. While we did not stratify by ICU subtype, our findings offer a general understanding of the relationship between glucose variability and outcomes across the ICU population. Lastly, the limited one-year follow-up for fatalities restricts long-term insights. Future research should consider prospective multi-center studies to validate these findings.

## Conclusion

The combination of SHR and GV is promising to facilitate early identifying IS critically ill patients at high-risk of mortality, especially non-DM IS patients. Moreover, advanced ML model presents stronger performance in prediction than traditional risk score, SHR and GV also shows their great importance in the ML model. This study provides new evidence in tailoring personalized blood glucose management strategies, with the goal of enhancing critical ill IS patient outcomes.

## Supplementary Information

Supplementary Material 1

## Data Availability

The datasets used in this study are available from the corresponding author on request or at https://physionet.org/content/mimiciv/after a standard process.
