# Supplementary material for: Joint impact of stress hyperglycaemic ratio and glycaemic variability in patients with ischaemic stroke and machine learning for mortality prediction
Source: BMC Neurol. 2025 Nov 25;25:484. doi: 10.1186/s12883-025-04510-z (PMC12649026; doi:10.1186/s12883-025-04510-z)
Supplement: Supplementary file 1 — Supplementary Material 1 [file 12883_2025_4510_MOESM1_ESM.docx]

Supplementary Table S1 Missing values of variables in this study

| Varialbes | Percentage of missing, n (%) |
| --- | --- |
| Height | 237 (31.6) |
| Heart rate | 1 (0.1) |
| Systolic blood pressure | 3 (0.4) |
| Diastolic blood pressure | 3 (0.4) |
| Pulse oxygen staturation | 1 (0.1) |
| Calcium | 50 (6.7) |
| Hemoglobin | 3 (0.4) |
| White blood cell counts | 2 (0.3) |
| Platelets | 5 (0.7) |

Supplementary Table S2 Outcomes of ischaemic stroke patients according to tertile levels of SHR

| Outcomes | Total | T1 | T2 | T3 | *P-value* |
| --- | --- | --- | --- | --- | --- |
| Mortality, n (%) |  |  |  |  |  |
| 30-day ICU mortality | 174 (23.2) | 45 (18.1) | 52 (20.2) | 77 (31.6) | 0.001 |
| 90-day ICU mortality | 222 (29.6) | 60 (24.2) | 64 (24.9) | 98 (40.2) | < 0.001 |
| 360-day ICU mortality | 265 (35.4) | 76 (30.6) | 80 (31.1) | 109 (44.7) | 0.001 |
| Hospital mortality | 121 (16.2) | 29 (11.7) | 40 (15.6) | 52 (21.3) | 0.014 |
| ICU mortality | 78 (10.4) | 15 (6.0) | 28 (10.9) | 35 (14.3) | 0.010 |
| Length of ICU stay, days | 4.4 (2.9, 4.6) | 4.1 (2.6, 7.7) | 4.5 (2.9, 7.1) | 4.8 (3.0, 7.9) | 0.264 |
| Length of hospital stay, days | 8.7 (5.2,14.7) | 7.4 (5.0, 14.1) | 8.8 (5.1, 13.2) | 9.3 (5.8, 15.6) | 0.261 |

Abbreviations: ICU, intensive care unit; SHR, stress hyperglycaemic ratio.

Supplementary Table S3 The associations of SHR and GV with 30-day ICU mortality in ischaemic stroke patients

|  |  | Model 1 | | Model 2 | | Model 3 | |
| --- | --- | --- | --- | --- | --- | --- | --- |
|  |  | HR (95% CI) | *P-value* | HR (95% CI) | *P-value* | HR (95% CI) | *P-value* |
| **Overall** | |  |  |  |  |  |  |
| SHR | Continuous SHR | 2.02 (1.50, 2.72) | < 0.001 | 1.50 (1.08, 2.09) | 0.016 | 1.37 (0.95, 1.99) | 0.094 |
|  | T1 | *Reference* |  | *Reference* |  | *Reference* |  |
|  | T2 | 1.15 (0.77, 1.72) | 0.485 | 1.23 (0.82, 1.84) | 0.317 | 1.33 (0.87, 2.03) | 0.189 |
|  | T3 | 1.96 (1.36, 2.83) | < 0.001 | 1.71 (1.17, 2.50) | 0.005 | 1.65 (1.09, 2.49) | 0.017 |
|  | *P for trend* |  | < 0.001 |  | 0.005 |  | 0.017 |
|  |  |  |  |  |  |  |  |
| GV | Continuous GV | 1.02 (1.02, 1.03) | < 0.001 | 1.01 (1.01, 1.02) | 0.002 | 1.01 (1.00, 1.02) | 0.027 |
|  | T1 | 1.07 (0.70, 1.62) | 0.757 | 1.33 (0.87, 2.02) | 0.190 | 1.63 (1.05, 2.53) | 0.031 |
|  | T2 | *Reference* |  | *Reference* |  | *Reference* |  |
|  | T3 | 2.24 (1.56, 3.22) | < 0.001 | 1.90 (1.30, 2.78) | 0.001 | 2.18 (1.44, 3.31) | < 0.001 |
|  | *P for trend* |  | < 0.001 |  | 0.001 |  | < 0.001 |
|  |  | |  |  |  |  |  |
| **Patients without diabetes mellitus** | |  |  |  |  |  |  |
| SHR | Continuous SHR | 3.09 (2.02, 4.73) | < 0.001 | 2.50 (1.56, 3.99) | < 0.001 | 2.22 (1.32, 3.74) | 0.003 |
|  | T1 | *Reference* |  | *Reference* |  | *Reference* |  |
|  | T2 | 1.50 (0.89, 2.53) | 0.128 | 1.62 (0.95, 2.76) | 0.075 | 2.00 (1.14, 3.50) | 0.016 |
|  | T3 | 2.83 (1.71, 4.67) | < 0.001 | 2.62 (1.57, 4.38) | < 0.001 | 2.93 (1.65, 5.19) | < 0.001 |
|  | *P for trend* |  | < 0.001 |  | < 0.001 |  | < 0.001 |
|  |  |  |  |  |  |  |  |
| GV | Continuous GV | 1.02 (1.01, 1.03) | < 0.001 | 1.01 (1.00, 1.02) | 0.013 | 1.01 (1.00, 1.03) | 0.049 |
|  | T1 | 1.17 (0.73, 1.88) | 0.516 | 1.54 (0.96, 2.49) | 0.076 | 1.96 (1.16, 3.29) | 0.012 |
|  | T2 | *Reference* |  | *Reference* |  | *Reference* |  |
|  | T3 | 2.62 (1.65, 4.15) | < 0.001 | 2.12 (1.30, 3.45) | 0.002 | 2.80 (1.66, 4.73) | < 0.001 |
|  | *P for trend* |  | < 0.001 |  | 0.002 |  | < 0.001 |
| **Patients with diabetes mellitus** | |  |  |  |  |  |  |
| SHR | Continuous SHR | 1.43 (0.88, 2.31) | 0.148 | 1.03 (0.61, 1.74) | 0.907 | 0.92 (0.47, 1.79) | 0.806 |
|  | T1 | *Reference* |  | *Reference* |  | *Reference* |  |
|  | T2 | 0.81 (0.41, 1.60) | 0.539 | 0.83 (0.42, 1.66) | 0.603 | 0.66 (0.29, 1.51) | 0.324 |
|  | T3 | 1.17 (0.67, 2.04) | 0.579 | 0.86 (0.48, 1.54) | 0.606 | 0.95 (0.47, 1.94) | 0.885 |
|  | *P for trend* |  | 0.565 |  | 0.606 |  | 0.797 |
|  |  |  |  |  |  |  |  |
| GV | Continuous GV | 1.03 (1.01, 1.04) | 0.002 | 1.02 (1.00, 1.04) | 0.058 | 1.02 (0.99, 1.05) | 0.134 |
|  | T1 | 0.74 (0.29, 1.88) | 0.530 | 0.79 (0.30, 2.05) | 0.622 | 0.91 (0.32, 2.59) | 0.861 |
|  | T2 | *Reference* |  | *Reference* |  | *Reference* |  |
|  | T3 | 1.90 (1.00, 3.60) | 0.049 | 1.52 (0.76, 3.03) | 0.240 | 1.46 (0.64, 3.34) | 0.368 |
|  | *P for trend* |  | 0.018 |  | 0.180 |  | 0.329 |

SHR: T1 ≤ 0.90; 0.90 < T2 ≤ 1.12; T3 > 1.12

GV: T1 ≤ 12.20%; 12.20% < T2 ≤ 20.14%; T3 > 20.14%

Model 1: Unadjusted

Model 2: Adjusted for age, sex, race, body mass index, systolic blood pressure, diastolic blood pressure, heart rate, saturation of peripheral oxygen, Simplified Acute Physiology Score, Sequential Organ Failure Assessment

Model 3: Model2 further adjusted hypertension, diabetes mellitus, myocardial infarction, peripheral vascular disease, congestive heart failure, chronic kidney disease, chronic pulmonary disease, liver disease, metastatic solid tumour, malignant cancer, renal replacement therapy, mechanical ventilation, vasopressor, antiplatelet agent, anticoagulant, statin, insulin, estimated glomerular filtration rate, white blood cell, haemoglobin, platelet, calcium, sodium, potassium.

Abbreviations: CI, confidence interval; GV, glycaemic variability; HR, hazard ratio; ICU, intensive care unit; SHR, stress hyperglycaemic ratio.

Supplementary Table S4 The associations of SHR and GV with 90-day ICU mortality in ischaemic stroke patients

|  |  | Model 1 | | Model 2 | | Model 3 | |
| --- | --- | --- | --- | --- | --- | --- | --- |
|  |  | HR (95% CI) | *P-value* | HR (95% CI) | *P-value* | HR (95% CI) | *P-value* |
| **Overall** | |  |  |  |  |  |  |
| SHR | Continuous SHR | 2.00 (1.53, 2.61) | <0.001 | 1.37 (1.02, 1.84) | 0.036 | 1.41 (1.01, 1.97) | 0.042 |
|  | T1 | *Reference* |  | *Reference* |  | *Reference* |  |
|  | T2 | 1.06 (0.75, 1.51) | 0.742 | 1.13 (0.79, 1.61) | 0.515 | 1.20 (0.82, 1.74) | 0.349 |
|  | T3 | 1.90 (1.38, 2.63) | <0.001 | 1.62 (1.16, 2.25) | 0.005 | 1.62 (1.13, 2.33) | 0.009 |
|  | *P for trend* |  | <0.001 |  | 0.004 |  | 0.008 |
|  |  |  |  |  |  |  |  |
| GV | Continuous GV | 1.02 (1.02, 1.03) | <0.001 | 1.02 (1.01, 1.02) | 0.001 | 1.01 (1.00, 1.02) | 0.007 |
|  | T1 | 0.87 (0.61, 1.26) | 0.466 | 1.08 (0.75, 1.56) | 0.687 | 1.26 (0.86, 1.85) | 0.246 |
|  | T2 | *Reference* |  | *Reference* |  | *Reference* |  |
|  | T3 | 1.94 (1.42, 2.65) | <0.001 | 1.59 (1.15, 2.21) | 0.005 | 1.75 (1.22, 2.50) | 0.002 |
|  | *P for trend* |  | <0.001 |  | 0.006 |  | 0.002 |
|  |  | |  |  |  |  |  |
| **Patients without diabetes mellitus** | |  |  |  |  |  |  |
| SHR | Continuous SHR | 2.85 (1.92, 4.24) | <0.001 | 2.16 (1.41, 3.32) | <0.001 | 2.23 (1.37, 3.64) | 0.001 |
|  | T1 | *Reference* |  | *Reference* |  | *Reference* |  |
|  | T2 | 1.26 (0.81, 1.97) | 0.299 | 1.36 (0.87, 2.13) | 0.181 | 1.55 (0.97, 2.49) | 0.070 |
|  | T3 | 2.40 (1.57, 3.67) | <0.001 | 2.18 (1.41, 3.36) | <0.001 | 2.40 (1.47, 3.91) | <0.001 |
|  | *P for trend* |  | <0.001 |  | <0.001 |  | <0.001 |
|  |  |  |  |  |  |  |  |
| GV | Continuous GV | 1.02 (1.02, 1.03) | <0.001 | 1.01 (1.00, 1.02) | 0.008 | 1.01 (1.00, 1.03) | 0.044 |
|  | T1 | 1.00 (0.67, 1.51) | 0.987 | 1.33 (0.88, 2.01) | 0.177 | 1.54 (0.98, 2.41) | 0.060 |
|  | T2 | *Reference* |  | *Reference* |  | *Reference* |  |
|  | T3 | 2.36 (1.59, 3.51) | <0.001 | 1.83 (1.20, 2.80) | 0.005 | 2.15 (1.37, 3.38) | 0.001 |
|  | *P for trend* |  | <0.001 |  | 0.005 |  | 0.001 |
| **Patients with diabetes mellitus** | |  |  |  |  |  |  |
| SHR | Continuous SHR | 1.56 (1.04, 2.34) | 0.033 | 1.15 (0.73, 1.81) | 0.553 | 1.03 (0.59, 1.82) | 0.917 |
|  | T1 | *Reference* |  | *Reference* |  | *Reference* |  |
|  | T2 | 0.78 (0.42, 1.47) | 0.446 | 0.80 (0.42, 1.51) | 0.485 | 0.63 (0.30, 1.33) | 0.225 |
|  | T3 | 1.34 (0.81, 2.20) | 0.255 | 1.00 (0.59, 1.70) | 0.999 | 1.03 (0.56, 1.90) | 0.923 |
|  | *P for trend* |  | 0.233 |  | 0.983 |  | 0.967 |
|  |  |  |  |  |  |  |  |
| GV | Continuous GV | 1.03 (1.01, 1.04) | <0.001 | 1.03 (1.01, 1.04) | 0.008 | 1.02 (1.00, 1.05) | 0.057 |
|  | T1 | 0.48 (0.20, 1.16) | 0.102 | 0.52 (0.21, 1.28) | 0.155 | 0.64 (0.25, 1.67) | 0.366 |
|  | T2 | *Reference* |  | *Reference* |  | *Reference* |  |
|  | T3 | 1.55 (0.91, 2.65) | 0.108 | 1.33 (0.74, 2.38) | 0.341 | 1.19 (0.60, 2.32) | 0.622 |
|  | *P for trend* |  | 0.034 |  | 0.227 |  | 0.540 |

SHR: T1 ≤ 0.90, 0.90 < T2 ≤ 1.12, T3>1.12

GV: T1 ≤ 12.20%, 12.20% < T2 ≤ 20.14%, T3 > 20.14%

Model 1: Unadjusted

Model 2: Adjusted for age, sex, race, body mass index, systolic blood pressure, diastolic blood pressure, heart rate, saturation of peripheral oxygen, Simplified Acute Physiology Score, Sequential Organ Failure Assessment

Model 3: Model2 further adjusted hypertension, diabetes mellitus, myocardial infarction, peripheral vascular disease, congestive heart failure, chronic kidney disease, chronic pulmonary disease, liver disease, metastatic solid tumor, malignant cancer, renal replacement therapy, mechanical ventilation, vasopressor, antiplatelet agent, anticogulant, statin, insulin, estimated glomerular filtration rate, white blood cell, hemoglobin, platelet, calcium, sodium, potassium.

Abbreviations: CI, confidence interval; GV, glycaemic variability; HR, hazard ratio; ICU, intensive care unit; SHR, stress hyperglycaemic ratio.

Supplementary Table S5 The associations of SHR and GV with 360-day ICU mortality in ischaemic stroke patients

|  |  | Model 1 | | Model 2 | | Model 3 | |
| --- | --- | --- | --- | --- | --- | --- | --- |
|  |  | HR (95% CI) | *P-value* | HR (95% CI) | *P-value* | HR (95% CI) | *P-value* |
| **Overall** | |  |  |  |  |  |  |
| SHR | Continuous SHR | 1.94 (1.50, 2.50) | <0.001 | 1.31 (0.99, 1.72) | 0.058 | 1.46 (1.07, 1.99) | 0.017 |
|  | T1 | *Reference* |  | *Reference* |  | *Reference* |  |
|  | T2 | 1.04 (0.76, 1.43) | 0.790 | 1.11 (0.81, 1.53) | 0.508 | 1.17 (0.84, 1.63) | 0.362 |
|  | T3 | 1.70 (1.27, 2.28) | <0.001 | 1.47 (1.08, 1.99) | 0.013 | 1.50 (1.08, 2.10) | 0.016 |
|  | *P for trend* |  | <0.001 |  | 0.012 |  | 0.016 |
|  |  |  |  |  |  |  |  |
| GV | Continuous GV | 1.02 (1.02, 1.03) | <0.001 | 1.01 (1.01, 1.02) | 0.001 | 1.01 (1.00, 1.02) | 0.020 |
|  | T1 | 0.83 (0.60, 1.16) | 0.279 | 1.03 (0.74, 1.43) | 0.862 | 1.18 (0.83, 1.67) | 0.350 |
|  | T2 | *Reference* |  | *Reference* |  | *Reference* |  |
|  | T3 | 1.83 (1.38, 2.43) | <0.001 | 1.49 (1.11, 2.01) | 0.009 | 1.51 (1.09, 2.09) | 0.014 |
|  | *P for trend* |  | <0.001 |  | 0.009 |  | 0.015 |
|  |  | |  |  |  |  |  |
| **Patients without diabetes mellitus** | |  |  |  |  |  |  |
| SHR | Continuous SHR | 2.50 (1.68, 3.71) | 0.000 | 1.96 (1.28, 3.00) | 0.002 | 2.33 (1.44, 3.76) | 0.001 |
|  | T1 | *Reference* |  | *Reference* |  | *Reference* |  |
|  | T2 | 1.22 (0.82, 1.81) | 0.328 | 1.32 (0.89, 1.98) | 0.170 | 1.44 (0.94, 2.20) | 0.091 |
|  | T3 | 2.04 (1.39, 3.01) | <0.001 | 1.92 (1.29, 2.85) | 0.001 | 2.16 (1.38, 3.38) | 0.001 |
|  | *P for trend* |  | <0.001 |  | 0.001 |  | 0.001 |
|  |  |  |  |  |  |  |  |
| GV | Continuous GV | 1.02 (1.02, 1.03) | <0.001 | 1.01 (1.00, 1.02) | 0.013 | 1.01 (1.00, 1.03) | 0.024 |
|  | T1 | 0.95 (0.65, 1.37) | 0.767 | 1.25 (0.86, 1.82) | 0.245 | 1.42 (0.95, 2.12) | 0.091 |
|  | T2 | *Reference* |  | *Reference* |  | *Reference* |  |
|  | T3 | 2.03 (1.40, 2.93) | <0.001 | 1.62 (1.10, 2.40) | 0.016 | 1.80 (1.18, 2.73) | 0.006 |
|  | *P for trend* |  | 0.001 |  | 0.016 |  | 0.005 |
| **Patients with diabetes mellitus** | |  |  |  |  |  |  |
| SHR | Continuous SHR | 1.63 (1.13, 2.34) | 0.009 | 1.11 (0.74, 1.66) | 0.623 | 1.01 (0.61, 1.67) | 0.967 |
|  | T1 | *Reference* |  | *Reference* |  | *Reference* |  |
|  | T2 | 0.82 (0.47, 1.42) | 0.471 | 0.83 (0.47, 1.46) | 0.513 | 0.74 (0.39, 1.40) | 0.352 |
|  | T3 | 1.29 (0.82, 2.02) | 0.269 | 0.97 (0.60, 1.56) | 0.896 | 0.99 (0.57, 1.73) | 0.973 |
|  | *P for trend* |  | 0.252 |  | 0.901 |  | 0.919 |
|  |  |  |  |  |  |  |  |
| GV | Continuous GV | 1.03 (1.01, 1.04) | <0.001 | 1.02 (1.00, 1.04) | 0.036 | 1.01 (0.99, 1.03) | 0.392 |
|  | T1 | 0.49 (0.23, 1.07) | 0.072 | 0.54 (0.24, 1.19) | 0.127 | 0.65 (0.27, 1.53) | 0.320 |
|  | T2 | *Reference* |  | *Reference* |  | *Reference* |  |
|  | T3 | 1.61 (0.99, 2.61) | 0.053 | 1.29 (0.76, 2.17) | 0.349 | 1.10 (0.60, 2.04) | 0.758 |
|  | *P for trend* |  | 0.012 |  | 0.226 |  | 0.650 |

SHR: T1 ≤ 0.90, 0.90 < T2 ≤ 1.12, T3>1.12

GV: T1 ≤ 12.20%, 12.20% < T2 ≤ 20.14%, T3 > 20.14%

Model 1: Unadjusted

Model 2: Adjusted for age, sex, race, body mass index, systolic blood pressure, diastolic blood pressure, heart rate, saturation of peripheral oxygen, Simplified Acute Physiology Score, Sequential Organ Failure Assessment

Model 3: Model2 further adjusted hypertension, diabetes mellitus, myocardial infarction, peripheral vascular disease, congestive heart failure, chronic kidney disease, chronic pulmonary disease, liver disease, metastatic solid tumor, malignant cancer, renal replacement therapy, mechanical ventilation, vasopressor, antiplatelet agent, anticogulant, statin, insulin, estimated glomerular filtration rate, white blood cell, hemoglobin, platelet, calcium, sodium, potassium.

Abbreviations: CI, confidence interval; GV, glycaemic variability; HR, hazard ratio; ICU, intensive care unit; SHR, stress hyperglycaemic ratio.

Supplementary Table S6 Subgroup analysis of SHR and 30-day ICU mortality

| Subgroups |  | HR (95% CI) | *P* | *P for interaction* |
| --- | --- | --- | --- | --- |
| Age < 70years | Q1 | Reference |  |  |
|  | Q2 | 1.83 (0.65, 5.12) | 0.252 |  |
|  | Q3 | 2.27 (0.88, 5.87) | 0.090 |  |
|  |  |  |  | 0.634 |
| Age ≥ 70years | Q1 | Reference |  |  |
|  | Q2 | 1.47 (0.90, 2.41) | 0.123 |  |
|  | Q3 | 1.70 (1.04, 2.77) | 0.034 |  |
|  |  |  |  |  |
| Male | Q1 | Reference |  |  |
|  | Q2 | 0.43 (0.20, 0.94) | 0.035 |  |
|  | Q3 | 0.74 (0.37, 1.50) | 0.404 |  |
|  |  |  |  | 0.711 |
| Female | Q1 | Reference |  |  |
|  | Q2 | 0.63 (0.36, 1.09) | 0.096 |  |
|  | Q3 | 0.85 (0.52, 1.41) | 0.536 |  |
|  |  |  |  |  |
| BMI < 30kg/m^2^ | Q1 | Reference |  |  |
|  | Q2 | 1.34 (0.80, 2.22) | 0.262 |  |
|  | Q3 | 2.17 (1.31, 3.57) | 0.002 |  |
|  |  |  |  | 0.563 |
| BMI ≥ 30kg/m^2^ | Q1 | Reference |  |  |
|  | Q2 | 1.54 (0.51, 4.58) | 0.442 |  |
|  | Q3 | 1.67 (0.59, 4.72) | 0.334 |  |

Abbreviations: BMI, body mass index; CI, confidence interval; HR, hazard ratio; ICU, intensive care unit; SHR, stress hyperglycaemic ratio.

Supplementary Table S7 Subgroup analysis of SHR and 90-day ICU mortality

| Subgroups |  | HR (95% CI) | *P* | *P for interaction* |
| --- | --- | --- | --- | --- |
| Age < 70years | Q1 | Reference |  |  |
|  | Q2 | 1.02 (0.43, 2.40) | 0.968 |  |
|  | Q3 | 1.96 (0.91, 4.21) | 0.086 |  |
|  |  |  |  | 0.242 |
| Age ≥ 70years | Q1 | Reference |  |  |
|  | Q2 | 1.51 (0.97, 2.33) | 0.067 |  |
|  | Q3 | 1.72 (1.11, 2.67) | 0.016 |  |
|  |  |  |  |  |
| Male | Q1 | Reference |  |  |
|  | Q2 | 1.50 (0.75, 2.97) | 0.250 |  |
|  | Q3 | 2.06 (1.05, 4.04) | 0.035 |  |
|  |  |  |  | 0.606 |
| Female | Q1 | Reference |  |  |
|  | Q2 | 1.21 (0.75, 1.98) | 0.437 |  |
|  | Q3 | 1.56 (0.96, 2.53) | 0.072 |  |
|  |  |  |  |  |
| BMI < 30kg/m^2^ | Q1 | Reference |  |  |
|  | Q2 | 1.14 (0.72, 1.79) | 0.578 |  |
|  | Q3 | 2.08 (1.33, 3.25) | 0.001 |  |
|  |  |  |  | 0.453 |
| BMI ≥ 30kg/m^2^ | Q1 | Reference |  |  |
|  | Q2 | 1.47 (0.63, 3.46) | 0.375 |  |
|  | Q3 | 1.45 (0.63, 3.33) | 0.377 |  |

Abbreviations: BMI, body mass index; CI, confidence interval; HR, hazard ratio; ICU, intensive care unit; SHR, stress hyperglycaemic ratio.

Supplementary Table S8 Subgroup analysis of SHR and 360-day ICU mortality

| Subgroups |  | HR (95% CI) | *P* | *P for interaction* |
| --- | --- | --- | --- | --- |
| Age < 70years | Q1 | Reference |  |  |
|  | Q2 | 1.08 (0.50, 2.35) | 0.848 |  |
|  | Q3 | 1.96 (0.95, 4.03) | 0.067 |  |
|  |  |  |  | 0.285 |
| Age ≥ 70years | Q1 | Reference |  |  |
|  | Q2 | 1.47 (0.99, 2.19) | 0.057 |  |
|  | Q3 | 1.62 (1.08, 2.44) | 0.019 |  |
|  |  |  |  |  |
| Male | Q1 | Reference |  |  |
|  | Q2 | 1.36 (0.75, 2.47) | 0.318 |  |
|  | Q3 | 1.89 (1.05, 3.39) | 0.033 |  |
|  |  |  |  | 0.697 |
| Female | Q1 | Reference |  |  |
|  | Q2 | 1.24 (0.80, 1.94) | 0.343 |  |
|  | Q3 | 1.40 (0.89, 2.20) | 0.142 |  |
|  |  |  |  |  |
| BMI < 30kg/m^2^ | Q1 | Reference |  |  |
|  | Q2 | 1.07 (0.72, 1.59) | 0.753 |  |
|  | Q3 | 1.91 (1.28, 2.85) | 0.002 |  |
|  |  |  |  | 0.363 |
| BMI ≥ 30kg/m^2^ | Q1 | Reference |  |  |
|  | Q2 | 1.66 (0.74, 3.73) | 0.222 |  |
|  | Q3 | 1.39 (0.63, 3.08) | 0.412 |  |

Abbreviations: BMI, body mass index; CI, confidence interval; HR, hazard ratio; ICU, intensive care unit; SHR, stress hyperglycaemic ratio.

Supplementary Table S9 Outcomes of ischaemic stroke patients according to tertile levels of GV

| Outcomes | Total | T1 | T2 | T3 | *P-value* |
| --- | --- | --- | --- | --- | --- |
| Mortality, n (%) |  |  |  |  |  |
| 30-day ICU mortality | 174 (23.2) | 44 (17.8) | 44 (17.3) | 86 (34.8) | < 0.001 |
| 90-day ICU mortality | 222 (29.6) | 53 (21.5) | 64 (25.1) | 105 (42.5) | < 0.001 |
| 360-day ICU mortality | 265 (35.4) | 64 (25.9) | 80 (31.4) | 121 (49.0) | < 0.001 |
| Hospital mortality | 121 (16.2) | 28 (11.3) | 33 (12.9) | 60 (24.3) | < 0.001 |
| ICU mortality | 78 (10.4) | 19 (7.7) | 17 (6.7) | 42 (17.0) | < 0.001 |
| Length of ICU stay, days | 4.4 (2.9, 7.6) | 3.7 (2.4, 5.1) | 4.3 (2.9, 7.9) | 5.9 (3.6, 11.5) | < 0.001 |
| Length of hospital stay, days | 8.7 (5.2, 14.7) | 6.8 (4.6, 10.1) | 9.5 (5.4, 15.7) | 11.0 (6.2, 18.3) | < 0.001 |

Abbreviations: ICU, intensive care unit; GV, glycaemic variability.

Supplementary Table S10 Subgroup analysis of GV and 30-day ICU mortality

| Subgroups |  | HR (95% CI) | *P* | *P for interaction* |
| --- | --- | --- | --- | --- |
| Age < 70years | Q1 | 1.72 (0.54, 5.48) | 0.358 |  |
|  | Q2 | Reference |  |  |
|  | Q3 | 4.75 (1.76, 12.85) | 0.002 |  |
|  |  |  |  | 0.126 |
| Age ≥ 70years | Q1 | 1.89 (1.14, 3.13) | 0.013 |  |
|  | Q2 | Reference |  |  |
|  | Q3 | 1.85 (1.13, 3.04) | 0.015 |  |
|  |  |  |  |  |
| Male | Q1 | 1.03 (0.46, 2.33) | 0.935 |  |
|  | Q2 | Reference |  |  |
|  | Q3 | 2.41 (1.15, 5.04) | 0.020 |  |
|  |  |  |  | 0.271 |
| Female | Q1 | 2.41 (1.35, 4.29) | 0.003 |  |
|  | Q2 | Reference |  |  |
|  | Q3 | 2.67 (1.50, 4.76) | 0.001 |  |
|  |  |  |  |  |
| BMI < 30kg/m^2^ | Q1 | 1.65 (0.96, 2.85) | 0.071 |  |
|  | Q2 | Reference |  |  |
|  | Q3 | 3.28 (1.98, 5.45) | <0.001 |  |
|  |  |  |  | 0.024 |
| BMI ≥ 30kg/m^2^ | Q1 | 2.81 (0.97, 8.14) | 0.060 |  |
|  | Q2 | Reference |  |  |
|  | Q3 | 1.39 (0.54, 3.60) | 0.495 |  |

Abbreviations: BMI, body mass index; CI, confidence interval; GV, glycemic variability; HR, hazard ratio; ICU, intensive care unit.

Supplementary Table S11 Subgroup analysis of GV and 90-day ICU mortality

| Subgroups |  | HR (95% CI) | *P* | *P for interaction* |
| --- | --- | --- | --- | --- |
| Age < 70years | Q1 | 1.81 (0.71, 4.61) | 0.216 |  |
|  | Q2 | Ref |  |  |
|  | Q3 | 3.58 (1.54, 8.33) | 0.003 |  |
|  |  |  |  | 0.286 |
| Age ≥ 70years | Q1 | 1.33 (0.85, 2.08) | 0.218 |  |
|  | Q2 | Ref |  |  |
|  | Q3 | 1.50 (0.98, 2.30) | 0.065 |  |
| Male | Q1 | 0.90 (0.45, 1.80) | 0.771 |  |
|  | Q2 | Ref |  |  |
|  | Q3 | 1.95 (1.05, 3.65) | 0.035 |  |
|  |  |  |  | 0.443 |
| Female | Q1 | 1.57 (0.96, 2.59) | 0.075 |  |
|  | Q2 | Ref |  |  |
|  | Q3 | 1.93 (1.19, 3.15) | 0.008 |  |
| BMI < 30kg/m^2^ | Q1 | 1.21 (0.76, 1.93) | 0.413 |  |
|  | Q2 | Ref |  |  |
|  | Q3 | 2.18 (1.41, 3.37) | <0.001 |  |
|  |  |  |  | 0.140 |
| BMI ≥ 30kg/m^2^ | Q1 | 1.88 (0.76, 4.68) | 0.174 |  |
|  | Q2 | Ref |  |  |
|  | Q3 | 1.41 (0.66, 3.01) | 0.380 |  |

Abbreviations: BMI, body mass index; CI, confidence interval; GV, glycemic variability; HR, hazard ratio; ICU, intensive care unit.

Supplementary Table S12 Subgroup analysis of GV and 360-day ICU mortality

| Subgroups |  | HR (95% CI) | *P* | *P for interaction* |
| --- | --- | --- | --- | --- |
| Age < 70years | Q1 | 1.45 (0.64, 3.27) | 0.369 |  |
|  | Q2 | Ref |  |  |
|  | Q3 | 2.65 (1.28, 5.48) | 0.009 |  |
|  |  |  |  | 0.181 |
| Age ≥ 70years | Q1 | 1.32 (0.88, 2.00) | 0.180 |  |
|  | Q2 | Ref |  |  |
|  | Q3 | 1.35 (0.91, 2.00) | 0.131 |  |
|  |  |  |  |  |
| Male | Q1 | 0.95 (0.51, 1.75) | 0.863 |  |
|  | Q2 | Ref |  |  |
|  | Q3 | 1.64 (0.95, 2.83) | 0.075 |  |
|  |  |  |  | 0.589 |
| Female | Q1 | 1.42 (0.89, 2.24) | 0.138 |  |
|  | Q2 | Ref |  |  |
|  | Q3 | 1.66 (1.06, 2.59) | 0.028 |  |
|  |  |  |  |  |
| BMI < 30kg/m^2^ | Q1 | 1.07 (0.71, 1.60) | 0.754 |  |
|  | Q2 | Ref |  |  |
|  | Q3 | 1.66 (1.13, 2.45) | 0.011 |  |
|  |  |  |  | 0.464 |
| BMI ≥ 30kg/m^2^ | Q1 | 2.13 (0.90, 5.01) | 0.085 |  |
|  | Q2 | Ref |  |  |
|  | Q3 | 1.64 (0.79, 3.40) | 0.187 |  |

Abbreviations: BMI, body mass index; CI, confidence interval; GV, glycemic variability; HR, hazard ratio; ICU, intensive care unit.

Supplementary Table S13 Best hyperparameters of each machine learning model

| Classifiers | Hyperparameters | |
| --- | --- | --- |
| Light gradient boosting machine | n_estimators | 143 |
|  | max_depth | 7 |
|  | learning_rate | 0.046049394341692654 |
|  | boosting_type | gbdt |
|  | objective | binary |
|  | num_leaves | 137 |
|  | colsample_bytree | 0.6427818028782419 |
|  | min_child_samples | 66 |
|  | subsample | 0.9261245937025091 |
|  | class_weight | 'balanced' |
|  |  |  |
| Random Forest | n_estimators | 112 |
|  | max_depth | 5 |
|  | criterion | ‘gini’ |
|  | min_samples_leaf | 10 |
|  | random_state | 3 |
|  | class_weight | ‘balanced’ |
|  |  |  |
| Logistic Regression | C | 10 |
|  | penalty | l2 |
|  | solver | ‘sag’ |
|  | max_iter | 100 |
|  | class_weight | ‘balanced’ |
|  |  |  |
| Support Vector Machine | C | 1 |
|  | kernel | ‘rbf’ |
|  | probability | True |
|  | tol | 0.001 |
|  | gamma | ‘auto’ |
|  | class_weight | ‘balanced’ |
|  |  |  |
| Multilayer Perceptron | solver | ‘adam’ |
|  | activation | ‘relu’ |
|  | hidden_layer_sizes | (10, 20, ) |
|  | alpha | 0.0001 |
|  | max_iter | 400 |
|  | random_state | 1 |
|  |  |  |
| K-nearest Neighbors | n_neighbors | 23 |
|  | p | 3 |
|  | metric | minkowski |
|  | weights | distance |

Supplementary Table S14 Sensitivity analysis of SHR and GV calculated based on average blood glucose levels within 48 hours in relation to mortality

|  |  | 30-day ICU mortality | | 90-day ICU mortality | | 360-day ICU mortality | |
| --- | --- | --- | --- | --- | --- | --- | --- |
|  |  | HR (95% CI) | *P-value* | HR (95% CI) | *P-value* | HR (95% CI) | *P-value* |
| **Overall** | |  |  |  |  |  |  |
| SHR | Continuous SHR | 2.29 (1.34, 3.91) | 0.002 | 2.23 (1.36, 3.65) | 0.001 | 2.30 (1.44, 3.65) | < 0.001 |
|  | T1 | *Reference* |  | *Reference* |  | *Reference* |  |
|  | T2 | 1.59(0.99, 2.54) | 0.052 | 1.39 (0.92, 2.11) | 0.117 | 1.21 (0.83, 1.75) | 0.330 |
|  | T3 | 2.59 (1.56, 4.27) | < 0.001 | 2.49 (1.61, 3.87) | < 0.001 | 2.31 (1.56, 3.42) | < 0.001 |
|  | *P for trend* |  | < 0.001 |  | 0.005 |  | 0.017 |
|  |  |  |  |  |  |  |  |
| GV | Continuous GV | 1.52 (1.02, 2.26) | 0.038 | 1.01 (1.04, 2.07) | 0.028 | 1.01 (0.99, 1.01) | 0.187 |
|  | T1 | 1.22 (0.91, 1.86) | 0.329 | 1.10 (0.76, 1.59) | 0.608 | 1.09 (0.77, 1.52) | 0.626 |
|  | T2 | *Reference* |  | *Reference* |  | *Reference* |  |
|  | T3 | 1.28 (1.03, 1.88) | 0.019 | 1.32 (1.05, 1.84) | 0.010 | 1.33 (1.02, 1.81) | 0.005 |
|  | *P for trend* |  | 0.037 |  | 0.021 |  | 0.008 |
| **Patients without diabetes mellitus** | |  |  |  |  |  |  |
| SHR | Continuous SHR | 3.25 (1.78, 5.92) | < 0.001 | 2.60 (1.53, 4.42) | < 0.001 | 2.63 (1.57, 4.39) | < 0.001 |
|  | T1 | *Reference* |  | *Reference* |  | *Reference* |  |
|  | T2 | 2.34 (1.27, 4.31) | 0.006 | 1.60 (0.96, 2.67) | 0.070 | 1.27 (0.81, 2.01) | 0.293 |
|  | T3 | 4.04 (2.20, 7.44) | < 0.001 | 3.07 (1.85, 5.10) | < 0.001 | 2.56 (1.63, 4.01) | < 0.001 |
|  | *P for trend* |  | <0.001 |  | < 0.001 |  | < 0.001 |
|  |  |  |  |  |  |  |  |
| GV | Continuous GV | 1.01 (0.99, 1.02) | 0.302 | 1.00 (0.99, 1.01) | 0.495 | 1.01 (0.99, 1.02) | 0.234 |
|  | T1 | 1.34 (0.81, 2.20) | 0.249 | 1.16 (0.75, 1.80) | 0.510 | 1.07 (0.72, 1.61) | 0.716 |
|  | T2 | *Reference* |  | *Reference* |  | *Reference* |  |
|  | T3 | 1.60 (0.96, 2.65) | 0.067 | 1.50 (0.98, 2.30) | 0.062 | 1.93 (0.84, 4.48) | 0.097 |
|  | *P for trend* |  | 0.067 |  | 0.172 |  | 0.223 |
| **Patients with diabetes mellitus** | |  |  |  |  |  |  |
| SHR | Continuous SHR | 2.36 (0.82, 6.85) | 0.113 | 2.16 (0.83, 5.63) | 0.116 | 1.01 (0.61, 1.67) | 0.124 |
|  | T1 | *Reference* |  | *Reference* |  | *Reference* |  |
|  | T2 | 0.92 (0.41, 2.03) | 0.829 | 0.88 (0.43, 1.81) | 0.738 | 0.84 (0.44, 1.61) | 0.616 |
|  | T3 | 2.03 (0.92, 4.47) | 0.085 | 1.95 (0.96, 3.95) | 0.064 | 1.92 (1.03, 3.61) | 0.041 |
|  | *P for trend* |  | 0.081 |  | 0.051 |  | 0.021 |
|  |  |  |  |  |  |  |  |
| GV | Continuous GV | 1.01 (0.99, 1.03) | 0.510 | 1.01 (0.99, 1.03) | 0.324 | 1.01 (0.98, 1.02) | 0.539 |
|  | T1 | 0.80 (0.32, 1.75) | 0.632 | 0.98 (0.45, 2.16) | 0.971 | 1.31 (0.65, 2.63) | 0.449 |
|  | T2 | *Reference* |  | *Reference* |  | *Reference* |  |
|  | T3 | 0.87 (0.44, 2.23) | 0.712 | 1.03 (0.54, 1.93) | 0.933 | 1.23 (0.68, 2.23) | 0.485 |
|  | *P for trend* |  | 0.878 |  | 0.993 |  | 0.706 |

SHR: T1 ≤ 0.92; 0.92 < T2 ≤ 1.09; T3 > 1.09

GV: T1 ≤ 9.16%; 9.16% < T2 ≤ 17.65%; T3 > 17.65%

Model: Adjusted for age, sex, race, body mass index, systolic blood pressure, diastolic blood pressure, heart rate, saturation of peripheral oxygen, Simplified Acute Physiology Score, Sequential Organ Failure Assessment, hypertension, diabetes mellitus, myocardial infarction, peripheral vascular disease, congestive heart failure, chronic kidney disease, chronic pulmonary disease, liver disease, metastatic solid tumour, malignant cancer, renal replacement therapy, mechanical ventilation, vasopressor, antiplatelet agent, anticoagulant, statin, insulin, estimated glomerular filtration rate, white blood cell, haemoglobin, platelet, calcium, sodium, potassium.

Abbreviations: CI, confidence interval; GV, glycaemic variability; HR, hazard ratio; ICU, intensive care unit; SHR, stress hyperglycaemic ratio.

Supplementary Table S15 Sensitivity analysis of the combination of SHR and GV calculated based on average blood glucose levels within 48 hours in relation to mortality

|  | 30-day ICU mortality | | 90-day ICU mortality | | 360-day ICU mortality | |
| --- | --- | --- | --- | --- | --- | --- |
|  | HR (95% CI) | *P-value* | HR (95% CI) | *P-value* | HR (95% CI) | *P-value* |
| **Overall** |  |  |  |  |  |  |
| G1 | *Reference* |  | *Reference* |  | *Reference* |  |
| G2 | 1.44 (0.91, 2.28) | 0.117 | 1.66 (1.09, 2.53) | 0.017 | 1.55 (1.06, 2.26) | 0.024 |
| G3 | 1.71 (1.07, 2.72) | 0.025 | 1.74 (1.17, 2.59) | 0.006 | 1.76 (1.23, 2.52) | 0.002 |
| G4 | 2.91 (1.87, 4.52) | < 0.001 | 2.73 (1.84, 4.04) | < 0.001 | 2.55 (1.77, 3.65) | < 0.001 |
| **Patients without diabetes mellitus** | | | | | | |
| G1 | *Reference* |  | *Reference* |  | *Reference* |  |
| G2 | 1.67 (0.92, 3.06) | 0.090 | 1.87 (1.13, 3.12) | 0.016 | 1.71 (1.01, 2.89) | 0.047 |
| G3 | 2.39 (1.27, 4.53) | 0.007 | 2.21 (1.26, 3.87) | 0.006 | 1.83 (1.15, 2.91) | 0.010 |
| G4 | 3.52 (2.05, 6.06) | < 0.001 | 3.14 (1.94, 4.07) | < 0.001 | 2.67 (1.70, 4.16) | < 0.001 |
| **Patients with diabetes mellitus** | | | | | | |
| G1 | *Reference* |  | *Reference* |  | *Reference* |  |
| G2 | 2.20 (0.85, 5.72) | 0.101 | 1.07 (0.51, 2.23) | 0.866 | 1.35 (0.70, 2.59) | 0.368 |
| G3 | 0.88 (0.40, 1.99) | 0.774 | 1.78 (0.82, 3.87) | 0.147 | 1.74 (0.85, 3.57) | 0.131 |
| G4 | 1.57 (0.65, 3.77) | 0.310 | 2.34 (1.05, 4.40) | 0.045 | 3.05 (1.44, 5.45) | 0.003 |

G1: Low SHR and low GV (SHR ≤ 1.09) and GV ≤ 17.65%); G2: High SHR and low GV (SHR > 1.09) and GV ≤ 17.65%); G3: Low SHR and high GV (SHR ≤ 1.09) and GV > 17.65%); G4: High SHR and high GV (SHR > 1.09) and GV > 17.65%).

Model: Adjusted for age, sex, race, body mass index, systolic blood pressure, diastolic blood pressure, heart rate, saturation of peripheral oxygen, Simplified Acute Physiology Score, Sequential Organ Failure Assessment, hypertension, diabetes mellitus, myocardial infarction, peripheral vascular disease, congestive heart failure, chronic kidney disease, chronic pulmonary disease, liver disease, metastatic solid tumour, malignant cancer, renal replacement therapy, mechanical ventilation, vasopressor, antiplatelet agent, anticoagulant, statin, insulin, estimated glomerular filtration rate, white blood cell, haemoglobin, platelet, calcium, sodium, potassium.

Abbreviations: CI, confidence interval; GV, glycaemic variability; HR, hazard ratio; ICU, intensive care unit; SHR, stress hyperglycaemic ratio.

Supplementary Table S16 Sensitivity analysis of the SD with mortality in ischaemic stroke patients

|  |  | 30-day ICU mortality | | 90-day ICU mortality | | 360-day ICU mortality | |
| --- | --- | --- | --- | --- | --- | --- | --- |
|  |  | HR (95% CI) | *P-value* | HR (95% CI) | *P-value* | HR (95% CI) | *P-value* |
| **Overall** | |  |  |  |  |  |  |
| SD | Continuous SD | 1.01 (0.99, 1.02) | 0.298 | 1.00 (0.99, 1.01) | 0.209 | 1.00 (0.99, 1.01) | 0.157 |
|  | T1 | *Reference* |  | *Reference* |  | *Reference* |  |
|  | T2 | 1.18 (0.76, 1.84) | 0.444 | 1.15 (0.80, 1.70) | 0.481 | 1.33 (0.87, 2.03) | 0.579 |
|  | T3 | 1.56 (1.06, 2.43) | 0.042 | 1.68 (1.13, 2.48) | 0.010 | 1.65 (1.09, 2.49) | 0.005 |
|  | *P for trend* |  | 0.124 |  | 0.021 |  | 0.008 |
| **Patients without diabetes mellitus** | |  |  |  |  |  |  |
| SD | Continuous SD | 3.09 (2.02, 4.73) | 0.183 | 1.00 (0.99, 1.01) | 0.401 | 1.00 (0.99, 1.01) | 0.186 |
|  | T1 | *Reference* |  | *Reference* |  | *Reference* |  |
|  | T2 | 1.21 (0.73, 1.98) | 0.459 | 1.22 (0.78, 1.89) | 0.390 | 1.20 (0.80, 1.80) | 0.377 |
|  | T3 | 1.48 (0.85, 2.57) | 0.160 | 1.70 (1.04, 2.79) | 0.034 | 1.77 (1.12, 2.78) | 0.014 |
|  | *P for trend* |  | 0.373 |  | 0.097 |  | 0.041 |
| **Patients with diabetes mellitus** | |  |  |  |  |  |  |
| SD | Continuous SD | 1.00 (0.99, 1.01) | 0.415 | 1.01 (0.99, 1.02) | 0.220 | 1.00 (0.99, 1.01) | 0.518 |
|  | T1 | *Reference* |  | *Reference* |  | *Reference* |  |
|  | T2 | 1.64 (0.52, 5.15) | 0.396 | 1.03 (0.40, 2.65) | 0.959 | 0.74 (0.32, 1.70) | 0.482 |
|  | T3 | 2.26 (0.85, 6.03) | 0.101 | 1.71 (0.78, 3.76) | 0.184 | 1.35 (0.70, 2.63) | 0.371 |
|  | *P for trend* |  | 0.231 |  | 0.221 |  | 0.194 |

SD: T1 ≤ 11.08mg/dl; 11.08mg/dl < T2 ≤ 23.67mg/dl; T3 > 23.67mg/dl

Model: Adjusted for age, sex, race, body mass index, systolic blood pressure, diastolic blood pressure, heart rate, saturation of peripheral oxygen, Simplified Acute Physiology Score, Sequential Organ Failure Assessment, hypertension, diabetes mellitus, myocardial infarction, peripheral vascular disease, congestive heart failure, chronic kidney disease, chronic pulmonary disease, liver disease, metastatic solid tumour, malignant cancer, renal replacement therapy, mechanical ventilation, vasopressor, antiplatelet agent, anticoagulant, statin, insulin, estimated glomerular filtration rate, white blood cell, haemoglobin, platelet, calcium, sodium, potassium.

Abbreviations: CI, confidence interval; HR, hazard ratio; ICU, intensive care unit; SD, standard deviation.

Supplementary Table S17 Sensitivity analysis of the quartiles of SHR and GV with mortality in ischaemic stroke patients

|  |  | 30-day ICU mortality | | 90-day ICU mortality | | 360-day ICU mortality | |
| --- | --- | --- | --- | --- | --- | --- | --- |
|  |  | HR (95% CI) | *P-value* | HR (95% CI) | *P-value* | HR (95% CI) | *P-value* |
| **Overall** | |  |  |  |  |  |  |
| SHR | Q1 | *Reference* |  | *Reference* |  | *Reference* |  |
|  | Q2 | 1.21 (0.75, 1.95) | 0.424 | 1.14 (0.75, 1.73) | 0.540 | 1.01 (0.69, 1.46) | 0.961 |
|  | Q3 | 1.45 (0.93, 2.32) | 0.114 | 1.32 (0.87, 1.99) | 0.194 | 1.21 (0.83, 1.75) | 0.318 |
|  | Q4 | 1.52 (1.09, 2.47) | 0.006 | 1.58 (1.04, 2.43) | 0.034 | 1.46 (1.05, 2.15) | 0.041 |
|  |  |  |  |  |  |  |  |
| GV | Q1 | 1.47 (0.91, 2.39) | 0.112 | 1.30 (0.85, 1.99) | 0.224 | 1.22 (0.83, 1.79) | 0.320 |
|  | Q2 | *Reference* |  | *Reference* |  | *Reference* |  |
|  | Q3 | 1.14 (0.72, 1.85) | 0.596 | 1.16 (0.76, 1.78) | 0.481 | 1.11 (0.76, 1.63) | 0.593 |
|  | Q4 | 1.90 (1.18, 3.19) | 0.008 | 1.93 (1.25, 2.97) | 0.003 | 1.62 (1.09, 2.41) | 0.018 |
| **Patients without diabetes mellitus** | |  |  |  |  |  |  |
| SHR | Q1 | *Reference* |  | *Reference* |  | *Reference* |  |
|  | Q2 | 1.57 (0.84, 2.96) | 0.159 | 1.39 (0.86, 2.39) | 0.237 | 1.14 (0.75, 1.73) | 0.540 |
|  | Q3 | 1.88 (1.02, 3.49) | 0.044 | 1.63 (0.96, 2.76) | 0.070 | 1.32 (0.87, 1.99) | 0.194 |
|  | Q4 | 2.61 (1.34, 5.09) | 0.005 | 2.17 (1.21, 3.91) | 0.009 | 1.58 (.04, 2.43) | 0.034 |
|  |  |  |  |  |  |  |  |
| GV | Q1 | 1.80 (1.01, 3.19) | 0.044 | 1.64 (0.99, 2.72) | 0.052 | 1.50 (0.95, 2.36) | 0.078 |
|  | Q2 | *Reference* |  | *Reference* |  | *Reference* |  |
|  | Q3 | 1.36 (0.75, 2.46) | 0.313 | 1.42 (0.85, 2.40) | 0.182 | 1.31 (0.82, 2.11) | 0.259 |
|  | Q4 | 2.73 (1.42, 5.23) | 0.003 | 2.85 (1.61, 5.07) | < 0.001 | 2.52 (1.47, 4.29) | < 0.001 |
| **Patients with diabetes mellitus** | |  |  |  |  |  |  |
| SHR | Q1 | *Reference* |  | *Reference* |  | *Reference* |  |
|  | Q2 | 0.79 (0.34, 1.86) | 0.593 | 0.68 (0.31, 1.46) | 0.325 | 0.68 (0.32, 1.46) | 0.325 |
|  | Q3 | 0.81 (0.35, 1.84) | 0.618 | 0.86 (0.40, 1.85) | 0.705 | 0.86 (0.40, 1.85) | 0.706 |
|  | Q4 | 1.07 (0.48, 2.39) | 0.864 | 1.06 (0.54, 2.09) | 0.869 | 1.06 (0.54, 2.09) | 0.869 |
|  |  |  |  |  |  |  |  |
| GV | Q1 | 0.89 (0.31, 2.63) | 0.845 | 0.65 (0.24, 1.74) | 0.385 | 0.65 (0.26, 1.60) | 0.350 |
|  | Q2 | *Reference* |  | *Reference* |  | *Reference* |  |
|  | Q3 | 0.82 (0.31, 2,17) | 0.691 | 0.67 (0.30, 1.49) | 0.322 | 0.68 (0.33, 1.40) | 0.296 |
|  | Q4 | 1.12 (0.44, 2.85) | 0.819 | 1.06 (0.48, 2.34) | 0.880 | 0.84 (0.41, 1.76) | 0.653 |

SHR: Q1 ≤ 0.86; 0.86 < Q2 ≤ 1.00; 1.00 < Q3 ≤ 1.18; Q4 > 1.18;

GV: Q1 ≤ 10.63%; 10.63% < Q2 ≤ 15.93%; 15.93% < Q3 ≤ 22.73%; Q4 > 22.73%.

Model: Adjusted for age, sex, race, body mass index, systolic blood pressure, diastolic blood pressure, heart rate, saturation of peripheral oxygen, Simplified Acute Physiology Score, Sequential Organ Failure Assessment, hypertension, diabetes mellitus, myocardial infarction, peripheral vascular disease, congestive heart failure, chronic kidney disease, chronic pulmonary disease, liver disease, metastatic solid tumour, malignant cancer, renal replacement therapy, mechanical ventilation, vasopressor, antiplatelet agent, anticoagulant, statin, insulin, estimated glomerular filtration rate, white blood cell, haemoglobin, platelet, calcium, sodium, potassium.

Abbreviations: CI, confidence interval; GV, glycaemic variability; HR, hazard ratio; ICU, intensive care unit; SHR, stress hyperglycaemic ratio.

Supplementary Table S18 Sensitivity analysis of the SHR and GV with mortality in ischaemic stroke patients after excluding glucose measurements < 3

|  |  | 30-day ICU mortality | | 90-day ICU mortality | | 360-day ICU mortality | |
| --- | --- | --- | --- | --- | --- | --- | --- |
|  |  | HR (95% CI) | *P-value* | HR (95% CI) | *P-value* | HR (95% CI) | *P-value* |
| **Overall** | |  |  |  |  |  |  |
| SHR | Continuous SHR | 1.46 (0.97, 2.19) | 0.065 | 1.48 (1.02, 2.15) | 0.037 | 1.56 (1.11, 2.20) | 0.011 |
|  | T1 | *Reference* |  | *Reference* |  | *Reference* |  |
|  | T2 | 1.76 (1.07, 2.91) | 0.027 | 1.51 (0.95, 2.39) | 0.075 | 1.48 (0.98, 2.23) | 0.061 |
|  | T3 | 2.26 (1.39, 3.68) | < 0.001 | 2.09 (1.34, 3.24) | 0.001 | 1.94 (1.30, 2.91) | 0.001 |
|  | *P for trend* |  | 0.004 |  | 0.004 |  | 0.005 |
|  |  |  |  |  |  |  |  |
| GV | Continuous GV | 1.01 (1.00, 1.02) | 0.043 | 1.01 (1.00, 1.02) | 0.021 | 1.01 (1.00, 1.02) | 0.043 |
|  | T1 | 1.69 (1.04, 2.77) | 0.034 | 1.42 (0.91, 2.22) | 0.118 | 1.41 (0.94, 2.11) | 0.092 |
|  | T2 | *Reference* |  | *Reference* |  | *Reference* |  |
|  | T3 | 1.96 (1.22, 3.15) | 0.005 | 1.59 (1.04, 2.44) | 0.031 | 1.43 (1.02, 2.12) | 0.039 |
|  | *P for trend* |  |  |  |  |  |  |
| **Patients without diabetes mellitus** | |  |  |  |  |  |  |
| SHR | Continuous SHR | 2.26 (1.23, 4.18) | 0.009 | 2.26 (0.93, 4.00) | 0.005 | 2.13 (1.23, 3.68) | 0.006 |
|  | T1 | *Reference* |  | *Reference* |  | *Reference* |  |
|  | T2 | 2.45 (1.28,4.70) | 0.007 | 1.86 (1.05, 3.30) | 0.034 | 1.69 (1.02, 2.82) | 0.041 |
|  | T3 | 4.03 (2.08, 7.80) | < 0.001 | 3.36 (1.87,6.05) | < 0.001 | 2.82 (1.67, 4,77) | < 0.001 |
|  | *P for trend* |  | 0.001 |  | < 0.001 |  | < 0.001 |
| GV | Continuous GV | 1.02 (1.01, 1.03) | 0.009 | 1.02 (1.00, 1.03) | 0.013 | 1.02 (1.01, 1.03) | 0.005 |
|  | T1 | 1.71 (0.96, 3.05) | 0.068 | 1.42 (0.84, 2.40) | 0.184 | 1.37 (0.86, 2.19) | 0.183 |
|  | T2 | *Reference* |  | *Reference* |  | *Reference* |  |
|  | T3 | 2.63 (1.48, 4.68) | < 0.001 | 2.09 (1.25, 3.51) | 0.005 | 1.83 (1.13, 2.95) | 0.014 |
|  | *P for trend* |  |  |  |  |  |  |
| **Patients with diabetes mellitus** | |  |  |  |  |  |  |
| SHR | Continuous SHR | 1.04 (0.37, 2.84) | 0.964 | 1.19 (0.53, 2.72) | 0.669 | 1.32 (0.66, 2.61) | 0.423 |
|  | T1 | *Reference* |  | *Reference* |  | *Reference* |  |
|  | T2 | 0.78 (0.22, 2.77) | 0.706 | 0.86 (0.29, 2.58) | 0.797 | 1.11 (0.43, 2.84) | 0.826 |
|  | T3 | 0.87 (0.31, 3.02) | 0.965 | 1.05 (0.29, 2.58) | 0.909 | 1.41 (0.63, 3.19) | 0.402 |
|  | *P for trend* |  | 0.924 |  | 0.940 |  | 0.695 |
|  |  |  |  |  |  |  |  |
| GV | Continuous GV | 1.04 (1.00, 1.08) | 0.047 | 1.04 (1.01, 1.08) | 0.014 | 1.01 (0.98, 1.04) | 0.356 |
|  | T1 | 1.35 (0.33, 5.52) | 0.680 | 1.16 (0.37, 4.04) | 0.809 | 1.83 (0.58, 5.76) | 0.300 |
|  | T2 | *Reference* |  | *Reference* |  | *Reference* |  |
|  | T3 | 1.75 (0.55, 5.55) | 0.342 | 1.35 (0.52, 3.54) | 0.539 | 1.24 (0.48, 3.22) | 0.658 |
|  | *P for trend* |  | 0.342 |  | 0.826 |  | 0.576 |

SHR: T1 ≤ 0.90; 0.90 < T2 ≤ 1.12; T3 > 1.12;

GV: T1 ≤ 12.22%; 12.22% < T2 ≤ 20.14%; T3 > 20.14%.

Model: Adjusted for age, sex, race, body mass index, systolic blood pressure, diastolic blood pressure, heart rate, saturation of peripheral oxygen, Simplified Acute Physiology Score, Sequential Organ Failure Assessment, hypertension, diabetes mellitus, myocardial infarction, peripheral vascular disease, congestive heart failure, chronic kidney disease, chronic pulmonary disease, liver disease, metastatic solid tumour, malignant cancer, renal replacement therapy, mechanical ventilation, vasopressor, antiplatelet agent, anticoagulant, statin, insulin, estimated glomerular filtration rate, white blood cell, haemoglobin, platelet, calcium, sodium, potassium.

Abbreviations: CI, confidence interval; GV, glycaemic variability; HR, hazard ratio; ICU, intensive care unit; SHR, stress hyperglycaemic ratio.

Supplementary Table S19 Sensitivity analysis of the combination of SHR and GV with mortality in ischaemic stroke patients after excluding glucose measurements < 3

|  | 30-day ICU mortality | | 90-day ICU mortality | | 360-day ICU mortality | |
| --- | --- | --- | --- | --- | --- | --- |
|  | HR (95% CI) | *P-value* | HR (95% CI) | *P-value* | HR (95% CI) | *P-value* |
| **Overall** |  |  |  |  |  |  |
| G1 | *Reference* |  | *Reference* |  | *Reference* |  |
| G2 | 3.58 (2.14, 5.96) | < 0.001 | 3.35 (2.10, 5.36) | < 0.001 | 3.25 (2.11, 4.98) | < 0.001 |
| G3 | 1.90 (1.08, 3.35) | 0.026 | 1.75 (1.05, 2.96) | 0.033 | 1.56 (0.97, 2.49) | 0.066 |
| G4 | 2.01 (1.17, 3.47) | 0.011 | 2.24 (1.37, 3.65) | 0.001 | 2.22 (1.42, 3.47) | < 0.001 |
| **Patients without diabetes mellitus** | | | | | | |
| G1 | *Reference* |  | *Reference* |  | *Reference* |  |
| G2 | 4.68 (2.52, 6.70) | < 0.001 | 4.27 (2.43, 7.51) | < 0.001 | 3.66 (2.19, 6.11) | < 0.001 |
| G3 | 3.17 (1.54, 6.55) | 0.002 | 2.80 (1.45, 5.38) | 0.002 | 2.11 (1.15, 3.86) | 0.016 |
| G4 | 2.65 (1.33, 5.40) | 0.006 | 2.63 (1.41, 4.93) | 0.002 | 2.41 (1.37, 4.22) | 0.002 |
| **Patients with diabetes mellitus** | | | | | | |
| G1 | *Reference* |  | *Reference* |  | *Reference* |  |
| G2 | 1.37 (0.34, 5.58) | 0.660 | 2.16 (0.60, 5.76) | 0.238 | 3.30 (1.38, 5.41) | 0.012 |
| G3 | 0.65 (0.22, 1.92) | 0.435 | 0.93 (0.34, 2.54) | 0.882 | 1.19 (0.48, 2.96) | 0.700 |
| G4 | 2.34 (0.69, 5.90) | 0.173 | 3.05 (1.04, 5.95) | 0.042 | 2.87 (1.06, 4.77) | 0.038 |

G1: Low SHR and low GV (SHR ≤ 1.12) and GV ≤ 20.14%); G2: High SHR and low GV (SHR > 1.12) and GV ≤ 20.14%); G3: Low SHR and high GV (SHR ≤ 1.12) and GV > 20.14%); G4: High SHR and high GV (SHR > 1.12) and GV > 20.14%).

Model: Adjusted for age, sex, race, body mass index, systolic blood pressure, diastolic blood pressure, heart rate, saturation of peripheral oxygen, Simplified Acute Physiology Score, Sequential Organ Failure Assessment, hypertension, diabetes mellitus, myocardial infarction, peripheral vascular disease, congestive heart failure, chronic kidney disease, chronic pulmonary disease, liver disease, metastatic solid tumour, malignant cancer, renal replacement therapy, mechanical ventilation, vasopressor, antiplatelet agent, anticoagulant, statin, insulin, estimated glomerular filtration rate, white blood cell, haemoglobin, platelet, calcium, sodium, potassium.

Abbreviations: CI, confidence interval; GV, glycaemic variability; HR, hazard ratio; ICU, intensive care unit; SHR, stress hyperglycaemic ratio.

Supplementary Table S20 The proportional hazards assumption was formally tested in your Cox regression models

|  | 30-day ICU mortality | 90-day ICU mortality | 360-day ICU mortality |
| --- | --- | --- | --- |
| **Overall** |  |  |  |
| Continuous SHR | 0.126 | 0.098 | 0.065 |
| SHR tertiles | 0.220 | 0.061 | 0.107 |
| Continuous GV | 0.113 | 0.069 | 0.110 |
| GV tertiles | 0.660 | 0.380 | 0.180 |
| **Patients without diabetes mellitus** | | | |
| Continuous SHR | 0.150 | 0.110 | 0.112 |
| SHR tertiles | 0.059 | 0.053 | 0.103 |
| Continuous GV | 0.410 | 0.260 | 0.120 |
| GV tertiles | 0.153 | 0.200 | 0.150 |
| **Patients with diabetes mellitus** | | | |
| Continuous SHR | 0.100 | 0.610 | 0.910 |
| SHR tertiles | 0.200 | 0.870 | 0.630 |
| Continuous GV | 0.430 | 0.350 | 0.770 |
| GV tertiles | 0.570 | 0.720 | 0.950 |

Abbreviations: CI, confidence interval; GV, glycaemic variability; ICU, intensive care unit; SHR, stress hyperglycaemic ratio


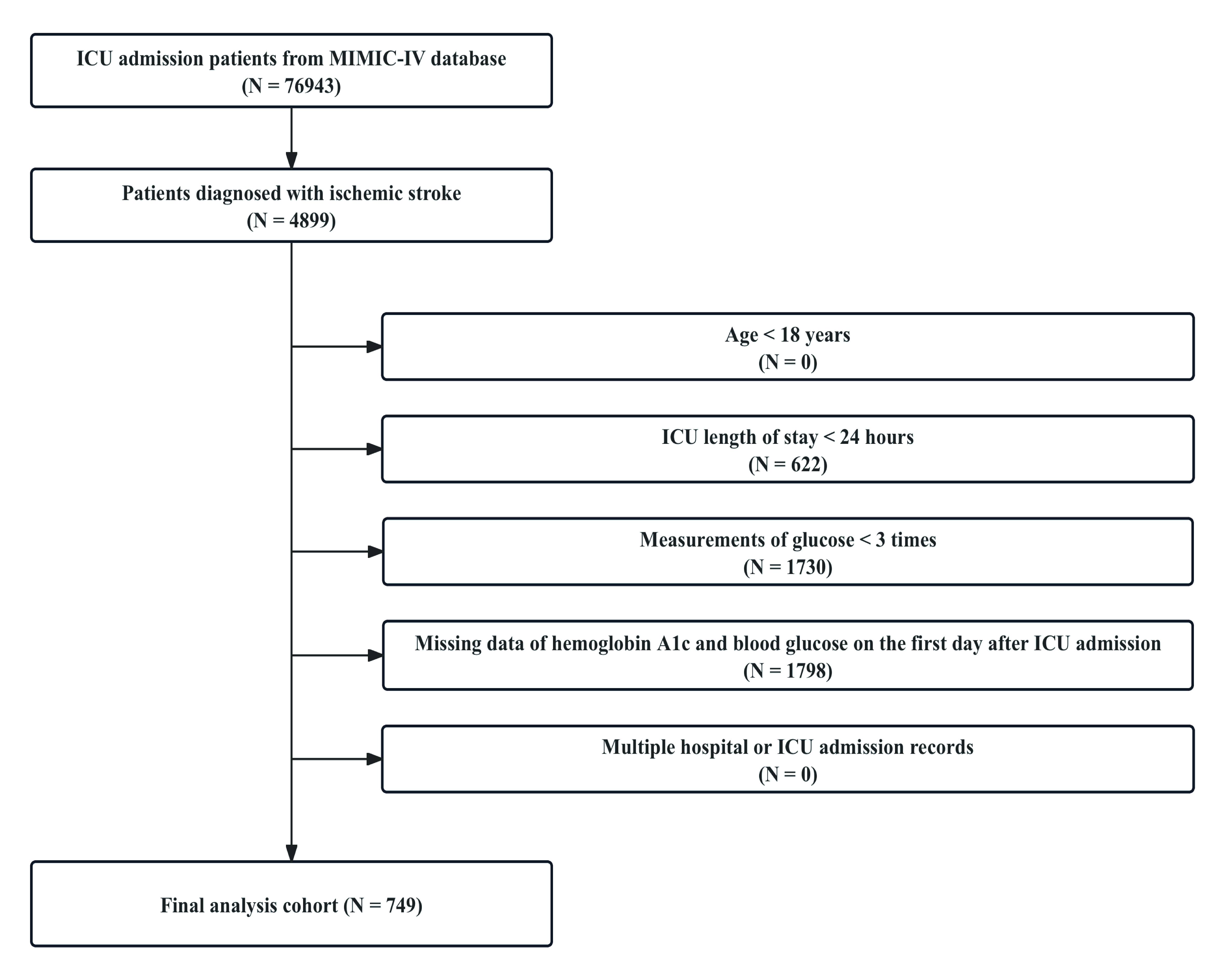


Supplementary Fig. S1 Flowchart of this study. Abbreviations: ICU, intensive care unit; MIMIC-IV, Medical Information Mart for Intensive Care-IV.

**
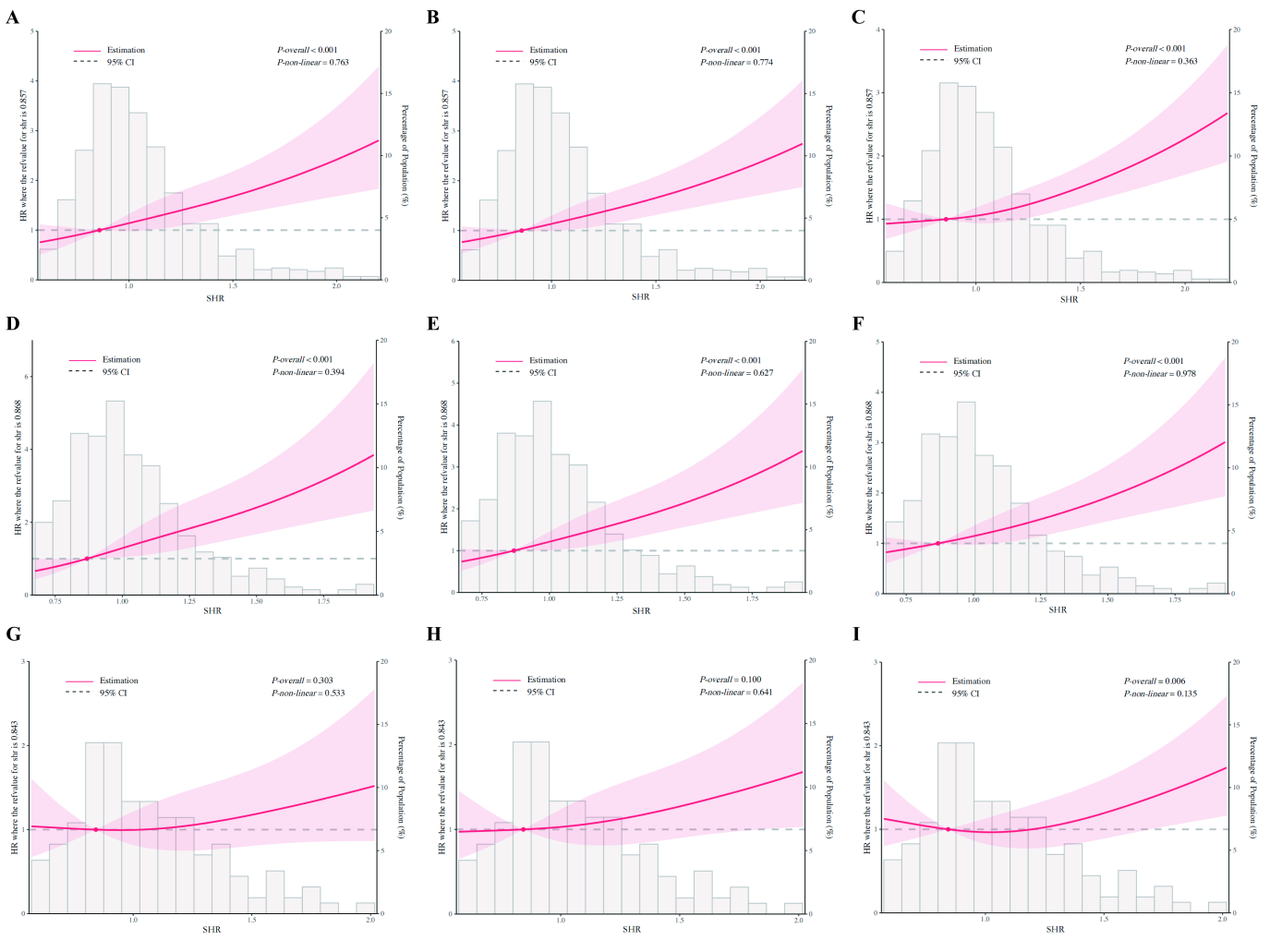
**

Supplementary Fig. S2 Multivariate-adjusted restricted cubic spline analyses of SHR for 30-day (A, D, G), 90-day (B, E, H), and 360-day (C, F, I) ICU mortality. Overall population (A-C); non-DM population (D-F); DM population (G-I). Abbreviations: CI, confidence interval; DM, diabetes mellitus; HR, hazard ratio; SHR, stress hyperglycemia ratio.


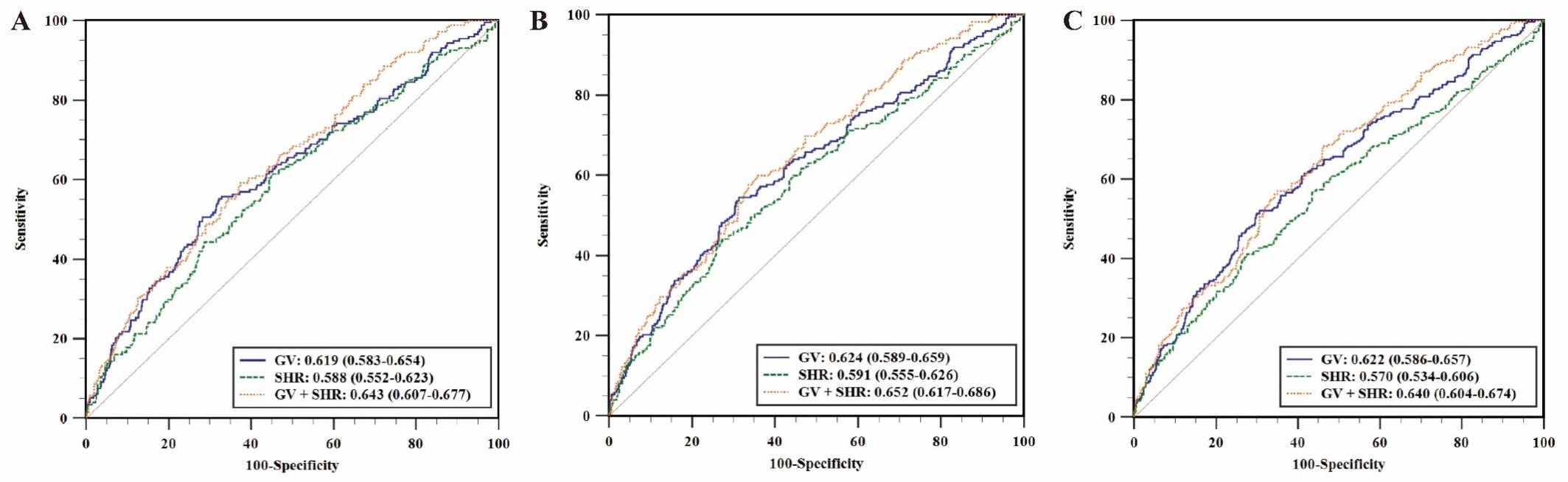


Supplementary Fig. S3 Receiver operating characteristic curves of SHR, GV, and their combination in predicting 30-day (A), 90-day (B), and 360-day (C) ICU mortality. Abbreviations: GV, glycemic variability; ICU, intensive care unit; SHR, stress hyperglycemia ratio.


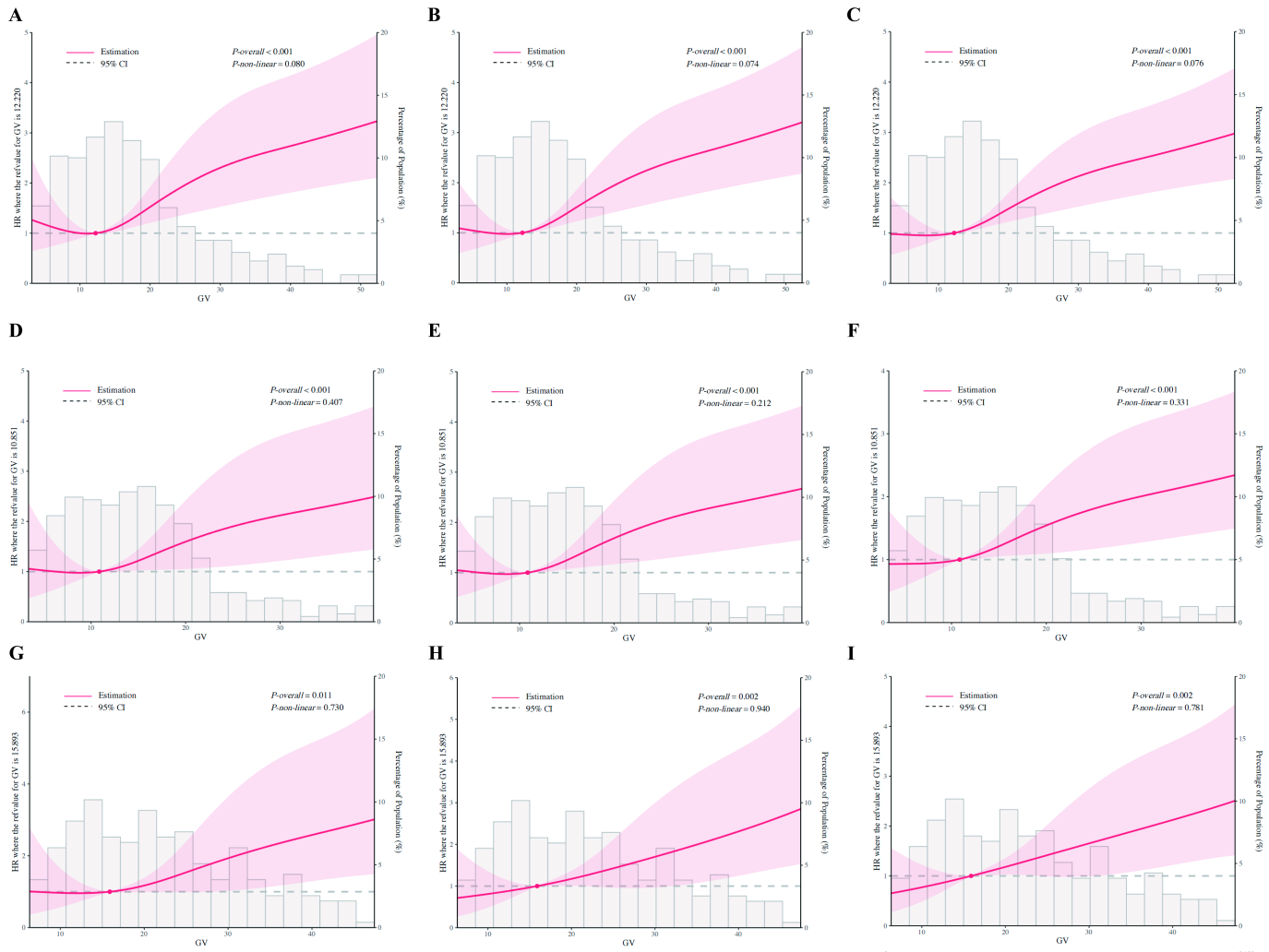


Supplementary Fig. S4 Multivariate-adjusted restricted cubic spline analyses of GV for 30-day (A, D, G), 90-day (B, E, H), and 360-day (C, F, I) ICU mortality. Overall population (A-C); non-DM population (D-F); DM population (G-I).





Supplementary Fig. S5 **Boruta feature importance ranking for predicting 30-day ICU all-cause mortality in patients with ischaemic stroke.** CHF, congestive heart failure; CKD, chronic kidney disease; CPD, chronic pulmonary disease; DBP, diastolic blood pressure; DM, diabetes mellitus; eGFR, estimated glomerular filtration rate; GV, glycaemic variability; HR, heart rate; ICU, intensive care unit; LD, liver disease; MC, malignant cancer; MI, myocardial infarction; MV, mechanical ventilation; MST, metastatic solid tumor; PVD, peripheral vascular disease; RRT, renal replacement therapy; SBP, systolic blood pressure; SHR, stress hyperglycaemic ratio; SPO2, saturation of peripheral oxygen; WBC, white blood cell.


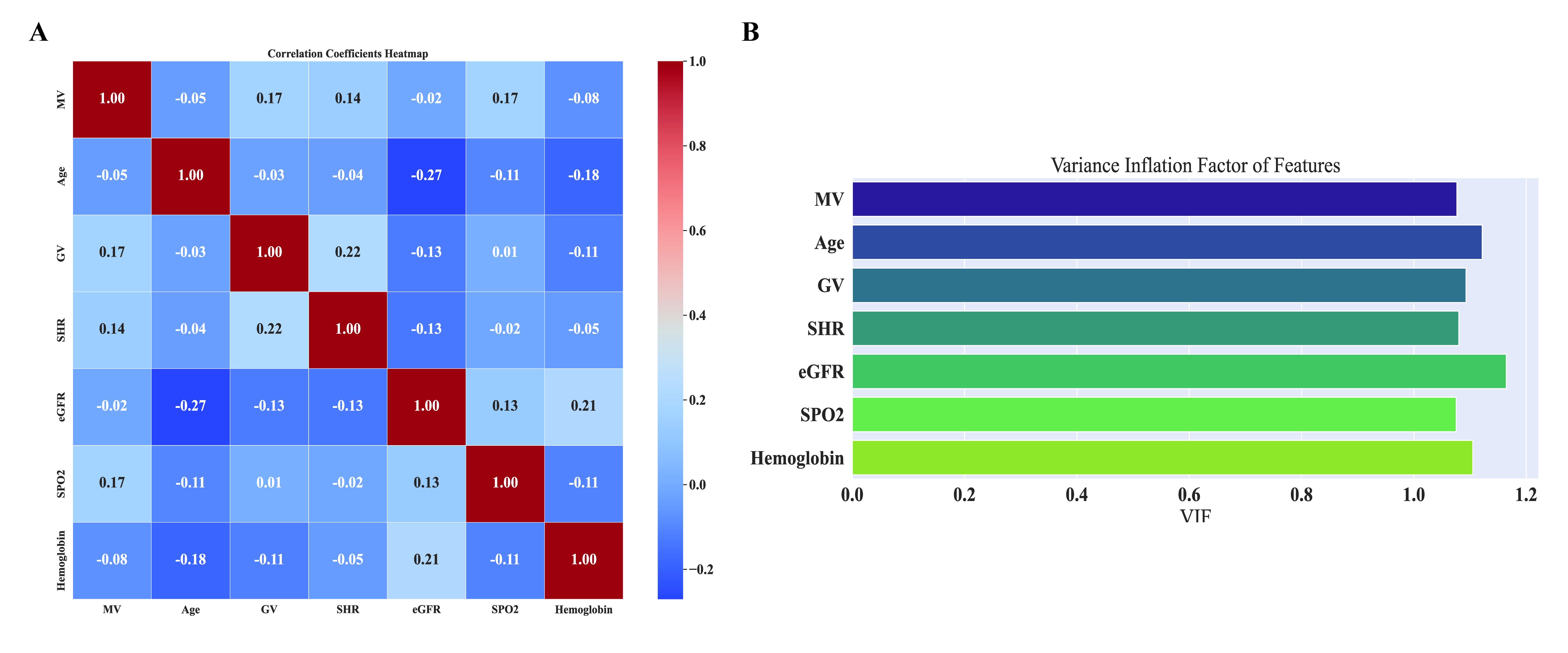


Supplementary Figure S6. Pearson's correlation test (A) and variance inflation factor test (B) for selected features.


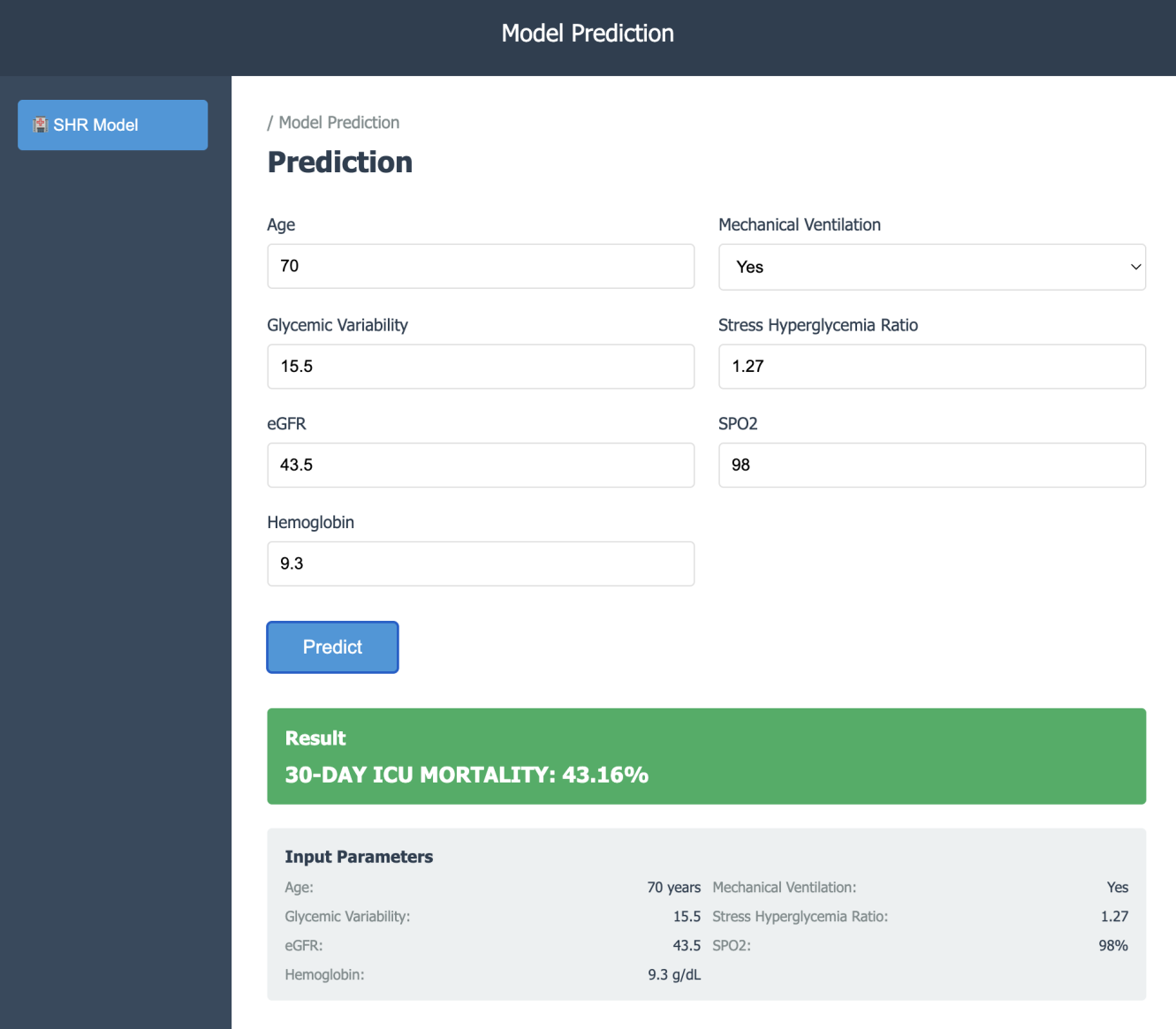


Supplementary Fig. S7 Web platform interface for the light gradient boosting machine model. eGFR, estimated glomerular filtration rate; GV, glycaemic variability; MV, mechanical ventilation; SHR, stress hyperglycaemic ratio.
